# Spatiotemporal variation and source analysis of air pollutants in the Harbin-Changchun (HC) region of China during 2014–2020

**DOI:** 10.1016/j.ese.2021.100126

**Published:** 2021-09-15

**Authors:** Yulong Wang, Youwen Sun, Zhiqing Zhang, Yuan Cheng

**Affiliations:** a State Key Laboratory of Urban Water Resource and Environment, School of Environment, Harbin Institute of Technology, Harbin, 150090, China; b Key Laboratory of Environmental Optics and Technology, Anhui Institute of Optics and Fine Mechanics, Chinese Academy of Sciences, Hefei, 230031, China

**Keywords:** Air pollution, Spatiotemporal variations, Harbin-changchun metropolitan area, Open biomass burning

## Abstract

This study analyzed the characteristics of air pollution and specific pollution periods within the Harbin-Changchun (HC) metropolitan area during 2014–2020. Regarding annual, seasonal, and monthly variations of the six pollutants, the change trend in 11 cities of HC showed strong consistency in spatial distribution. The western cities (Songyuan, Daqing, and Siping) were vulnerable to dust storms from Inner Mongolia. The concentrations of all air pollutants, except O_3_-8h, showed downward fluctuation trends from 2014 to 2018 and remained stable from 2018 to 2020 in terms of annual variations. The inter-annual trend of significant reductions in SO_2_ and SO_2_/PM_2.5_ during the heating period indicated that strict emission reduction measures posed by the government were highly successful. The ratio of PM_2.5_/SO_2_ was used to identify open biomass burning (OBB), which showed a double peak (October–November (Oct–Nov), March–April (Mar–Apr)). The burning prohibition shifted the OBB from Oct–Nov to Mar–Apr.

## Highlights

• Long-term records of air pollution were presented for Harbin-Changchun (HC) region.
• PM_2.5_ tended to decrease whereas O_3_ concentrations were largely unchanged.
• SO_2_ exhibited an apparent decreasing trend for the HC region.
• Open burning activities remained evident despite strict bans on agricultural fires.
• The western cities in HC were strongly impacted by dust during spring.

## 1 Introduction

As China's economy has grown, air pollution has been given greater attention by numerous studies [[1], [2], [3]]. To control air pollution problems, the government formulated a new air quality standard (GB3095-2012) in 2012. The “Air Pollution Prevention and Control Action Plan” issued by the State Council of China in 2013 was used to improve the air quality in China [4]. The implementation of the Action Plan has resulted in 121 of China's 338 cities meeting the new standards according to the “China Ecological Environment Status Bulletin in 2018” [5]. Moreover, many studies focus on metropolitan areas, such as the Beijing Tianjin Hebei (BTH; [[6], [7]], Yangtze River Delta (YRD; [1,8], and Pearl River Delta (PRD) regions [9] when studying air pollution. However, pollution mechanisms are highly variable due to different pollution sources and meteorological conditions within different regions [[10], [11], [12]]. The Harbin-Changchun (HC) metropolitan area located in Northeast China, has characteristics of a complex industrial and agricultural infrastructure, as well as unique climate characteristics [4,13,14].

HC is the only national-level city cluster in China that experiences an extremely long heating period [15]. During the heating period, a large number of pollutants are emitted due to the burning of fossil fuels [16,17]. Furthermore, HC is one of the most productive urban clusters in China in terms of grain yield [18] due to the large amount of “black land” according to the “China Statistical Yearbook” [19]. Furthermore, farmers burn the residue after the autumn harvest leading to haze episodes [[20], [21], [22], [23], [24]]. To control the haze episodes during this period, the local government carried out a burn ban to forbid open biomass burning (OBB; [[25], [26], [27]]. Moreover, dust storms are also a common air pollution event in HC [25]. The formation of air pollution in different cities has been influenced by local emission sources and climate characteristics [28,29]. At present, most of the existing studies are conducted during specific time periods within a city, and few studies are focused on high spatial and temporal resolution [30].

This study analyzed the spatiotemporal variations of the six air pollutants in HC during 2014–2020. The ratio of PM_10_ to PM_2.5_ and the back-trajectory analysis were used to discuss the influence of spring dust on different cities of HC. The influence of OBB on HC was analyzed by using the characteristic ratio of PM_2.5_ to SO_2_ and fire points. By analyzing the spatiotemporal variations of air pollutants and special pollution periods (spring dust period and OBB period), the state of air pollution in the HC region could be better understood.

## 2 Materials and methods

### 2.1 Study areas

**Fig. 1:**
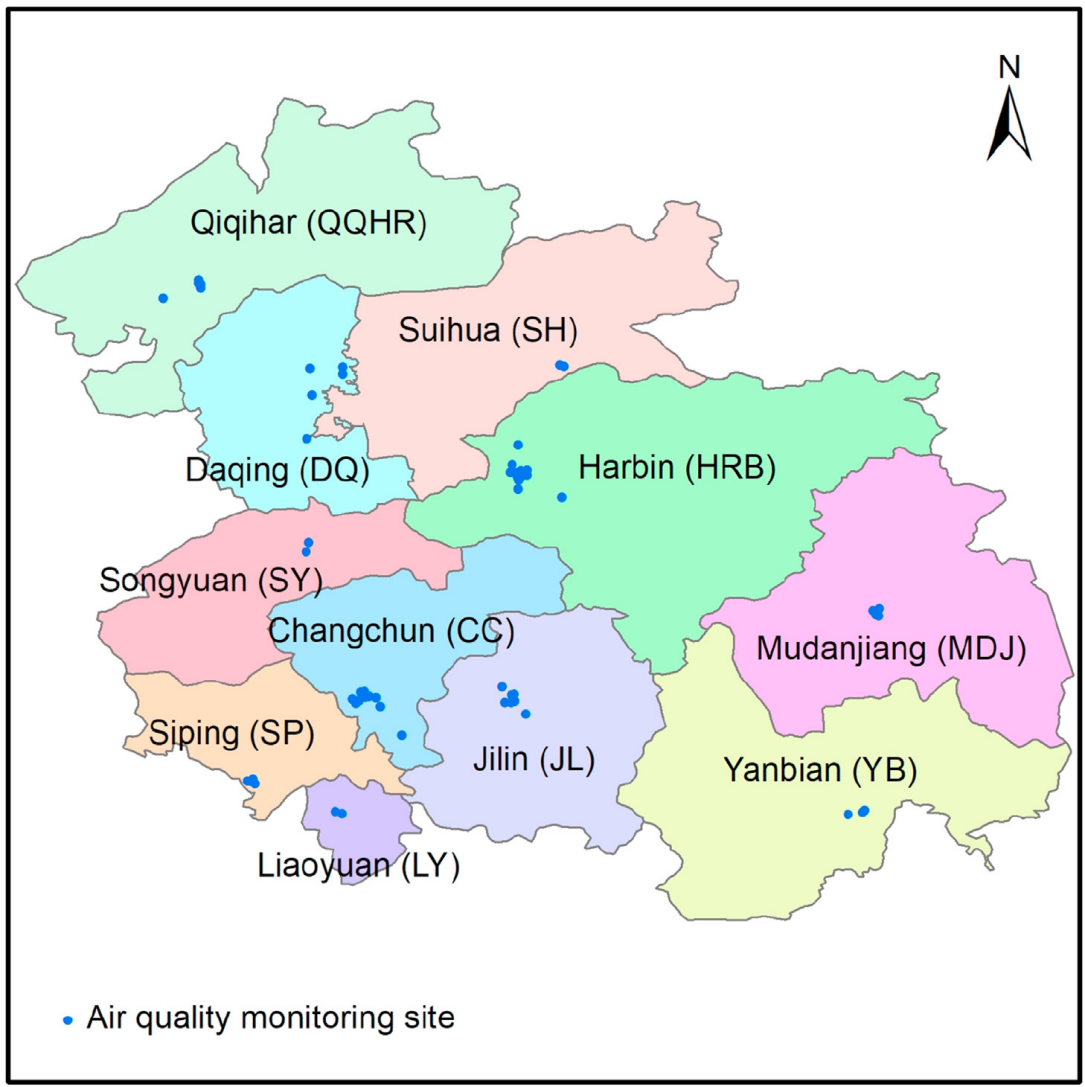
The geographical location of 11 cities and 58 air quality monitoring sites in HC region.

The HC region is located in Northeast China, including 5 cities (Harbin, Daqing, Qiqihar, Suihua, and Mudanjiang) in the Heilongjiang province and 6 cities (Changchun, Jilin, Siping, Liaoyuan, Songyuan, and Yanbian) in the Jilin province. The HC urban agglomeration has unique regional characteristics due to its six-month-long heating period and developed agriculture. In this work, the 11 cities (Fig. 1) mentioned above were selected in order to analyze the air pollution within the HC region.

### 2.2 Data source

Mass concentration data of six air pollutants (PM_2.5_, PM_10_, SO_2_, NO_2_, CO, and O_3_-8h) from 2014 to 2020 was downloaded from the China Air Quality Online Monitoring and Analysis Platform (https://www.aqistudy.cn/). O_3_-8h is the average O_3_ concentration during an 8 h time period in a single day. The detailed monitoring technology of the target pollutants is shown in the Supporting Information (SI-1), and can also be found within previous studies [1,26,31]. The original data of the six air pollutants were taken from 58 air quality monitoring stations (Fig. 1) within the HC region. In this study, the six air pollutants from 2014 to 2020 in 11 cities were analyzed in order to discuss spatiotemporal variations and unique pollution periods within the HC region.

### 2.3 Fire point

MODIS active fire products (C6) provided fire points from the Terra and Aqua satellites (https://firms.modaps.eosdis.nasa.gov/map/). MODIS C6 has a horizontal resolution of about 1 km^2^ and a temporal resolution of 4 times/day, which has been widely used to observe the fire points caused by OBB in Northeast China [24,32].

### 2.4 Backward trajectory and cluster analyses

To analyze the origin of the primary air mass source, the Hybrid Single-Particle Lagrangian Integrated Trajectory Model (HYSPLIT; https://www.ready.noaa.gov/HYSPLIT.php) which was provided by the USA National Oceanic and Atmospheric Administration (NOAA), was used to identify the direction of the spring dust. A two-dimensional 48h air mass backward trajectory, arriving every day (04:00 UTC) at 100 m above ground level in Songyuan (45°10′N, 124°48′E) was calculated using the GIS-based software TrajStat [33]. The HYSPLIT model used cluster analysis to determine the pollutants' primary source location and pollutant composition.

## 3 Results and discussion

### 3.1 Spatiotemporal variations of air pollutants

#### 3.1.1 Spatial distribution of air pollutants

According to the map in Fig. 1, Qiqihar, Daqing, and Suihua are located in the northern part of HC. The city of Jilin and the provincial capitals of Harbin and Changchun are located in the central area of the HC region. Songyuan, Siping, and Liaoyuan are located in the western part of HC, bordering Liaoning and Inner Mongolia. Mudanjiang and Yanbian are located in the eastern region of HC, bordering North Korea and Russia. This section investigates the long-term time series daily average levels of six air pollutants in 11 cities within the HC region. In terms of the spatial distribution of the air pollutants, the concentration of PM_2.5_, PM_10_, SO_2,_ and NO_2_ in the central cities (Jilin, Harbin, and Changchun) were higher than in the other cities of HC. Among the 11 cities, the concentration of CO is not significant within the spatial distribution of the pollutants (slightly higher in the central cities). In addition, there were no significant differences in the concentration of O_3_-8h among the 11 cities. In terms of annual, seasonal, and monthly variations of the six air pollutants, the 11 cities showed strong spatial distribution consistency.

#### 3.1.2 Annual variation of air pollutants

**Fig. 2:**
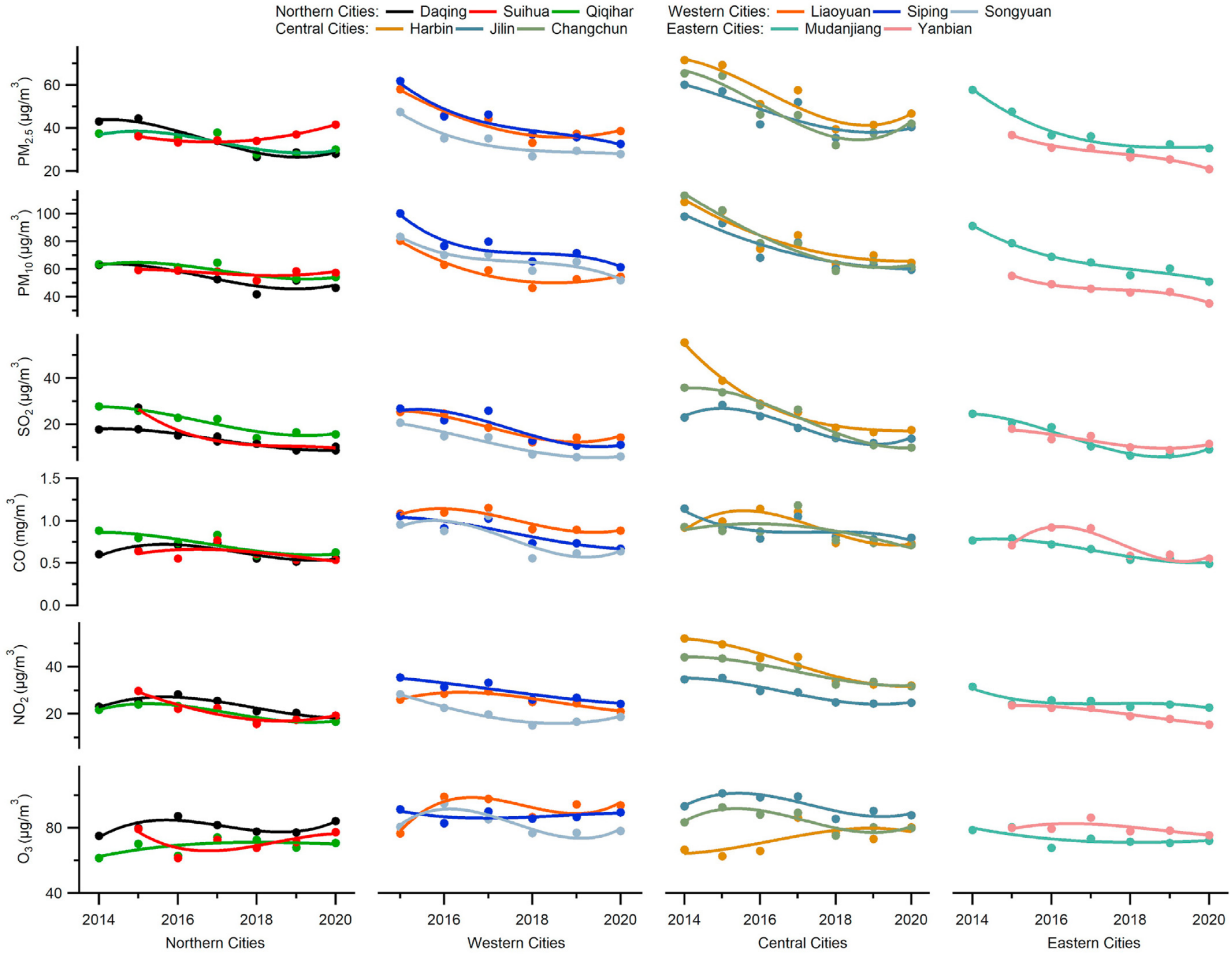
The annual trend analysis of six criteria pollutants (PM_2.5_, PM_10_, SO_2_, CO, NO_2_ and O_3_-8h) in 11 cities of HC during 2014–2020. The units of mass concentrations are μg/m^3^ for PM_2.5_, PM_10_, SO_2_, NO_2_, O_3_-8h, and mg/m^3^ for CO.

The inter-annual variation of the annual mean concentration and the annual trend analysis of the six air pollutants in the HC region during 2014–2020 are displayed in Fig. 2 and the annual average data of each pollutant in 11 cities of HC is shown in Table S1. According to the results of Fig. 2, the central cities (Jilin, Harbin, and Changchun) show higher levels of air pollutants. Northern cities (Qiqihar, Daqing, and Suihua), eastern cities (Songyuan, Siping, and Liaoyuan), and the western cities (Mudanjiang and Yanbian) have comparable pollution levels. As displayed in Fig. 2, the concentrations of PM_2.5_, PM_10_, SO_2_, NO_2,_ and CO showed downward fluctuation trends from 2014 to 2018 and remained stable from 2018 to 2020 in all cities (except Suihua). Fig. 2 shows that the concentrations of PM_2.5_ in Suihua remain stable between 2015 and 2018, with a slight upward trend from 2018 to 2020. The comparison results of the annual variation of PM_2.5_ in Beijing, Shanghai, Guangzhou, and Harbin are shown in Fig. S1. The data of PM_2.5_ in Beijing, Shanghai, and Guangzhou were taken from Ref. [34]; who also used the China Air Quality Online Monitoring and Analysis Platform data. Compared with Beijing, Shanghai, and Guangzhou, PM_2.5_'s rate of decrease in Harbin was slow. In terms of the annual variations of SO_2_, NO_2,_ and CO concentrations, the city of Suihua shows a similar regularity to other cities as shown in Fig. 2. The concentration of SO_2_ displayed a significant downward trend, especially in the provincial capitals Harbin and Changchun. The concentrations of SO_2_ in Harbin and Changchun reduced from 55.43 to 35.87 μg/m^3^ in 2014 to 17.42 and 9.91 μg/m^3^ in 2020. The significant decrease in SO_2_ in each city may be related to the government's energy conservation and emission reduction measures. The annual CO concentration trend is not entirely consistent across the cities; however, it shows a decreasing trend according to Fig. 2. The concentration of NO_2_ also shows a decreasing trend, but the decrease is smaller to that of SO_2_. Compared to other cities, the annual variation of NO_2_ concentration in the central cities Jilin, Harbin, and Changchun had the most significant decrease. The concentrations of NO_2_ in Jilin, Harbin, and Changchun have reduced from 34.66, 52.09, and 44.10 μg/m^3^ in 2014 to 24.72, 32.02, and 31.71 μg/m^3^ in 2020, respectively. The concentrations of O_3_-8h shown in Fig. 2 have a slight upward trend in Harbin, Qiqihar, Daqing, and Liaoyuan. Furthermore, the annual variations of the concentrations of O_3_-8h are stable in other cities of HC. In general, the trend of the six air pollutants in 11 cities of the HC region is similar in terms of annual variations.

#### 3.1.3 Season variation of air pollutants

The inter-annual variability of the seasons within the 11 cities of HC was investigated in Fig. S2, and was used to fully understand the temporal and spatial variations of the six air pollutants. The seasonal trend analysis of the six criteria pollutants in HC during 2014–2020 is displayed in Fig. S3. The results showed that the seasonal variation trend of the air pollutants within the 11 cities was similar; however, the pollution level is quite different. The concentration of PM_2.5_ was the lowest during the summer in all cities and was the highest in winter, except for in Suihua, Siping, and Daqing in 2015, which was the highest in the fall. The seasonal trends of PM_10_ were similar to PM_2.5_ in most cities. It is worth noting that the concentration of PM_10_ in Songyuan and Siping in the spring was significantly higher than in the other seasons. The reason may be that Songyuan and Siping are more vulnerable to sandstorms from Inner Mongolia during the spring, and is discussed in the next section. The concentrations of PM_2.5_ and PM_10_ decreased significantly in fall compared with other seasons from 2014 to 2018, which may be related to the government's emission reduction measures. According to the previous report, SO_2_ primarily comes from burning fossil fuels such as coal [35]. The concentration of SO_2_ was the highest in the winter and the lowest during the summer in all cities. As shown in Fig. S3, the concentration of SO_2_ in Harbin and Changchun is 6.14 and 8.07 μg/m^3^ in the summer and 49.78 and 60.63 μg/m^3^ in the winter, respectively. It is worth noting that the concentration of SO_2_ in the winter shows a rapidly decreasing trend in terms of inter-annual variation in all cities within the HC. The annual variation of the ratio of SO_2_ to PM_2.5_ also decreased significantly during the heating period (from October 15 to April 15 of the following year), according to Fig. S4. This phenomenon may be due to the government's emission reduction measures, such as eliminating small coal-fired boilers, which was also reported by Refs. [3,23,25,26]. The interannual variation of the NO_2_/SO_2_ ratio during the heating period within the 11 cities is displayed in Fig. S5, showing a trend of first increasing and then maintaining a stable NO_2_/SO_2_ ratio. This result shows that the government's emission reduction measures are more effective in controlling SO_2_ than NO_2_. As shown in Fig. S2 and Fig. S3, the seasonal variation trend of CO was the highest in winter and the lowest in summer. The seasonal variation trend of O_3_-8h showed an opposite trend in PM_2.5_ and PM_10_ concentrations, which were generally the lowest in winter and the highest in summer in all cities. In general, the seasonal variation of the PM_2.5_, PM_10_, SO_2_, NO_2,_ and CO concentrations are usually lowest in summer and highest in winter. The concentrations of O_3_-8h showed an opposite trend to the other five pollutants, with the lowest in winter and the highest in summer.

#### 3.1.4 Monthly variation of air pollutants

**Fig. 3:**
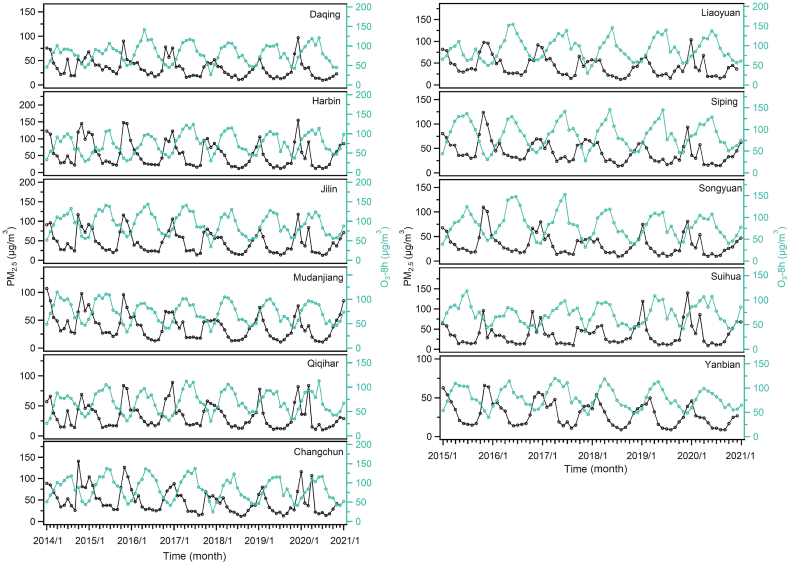
The monthly average mass concentrations of PM_2.5_ and O_3_-8h in 11 cities of HC. The units are μg/m^3^.

The monthly variation trends of the six air pollutant concentrations within the 11 cities were displayed in Fig. 3 and Fig. S6. The Pearson Correlation in different months within the 11 cities is displayed in Table. S2. The monthly variation trend and the Pearson Correlation showed that the concentrations of PM_2.5_, PM_10_, SO_2_, NO_2,_ and CO have similar monthly trends, while the concentration of O_3_-8h shows an opposite trend to the other five pollutants. The concentration of PM_2.5_, PM_10_, SO_2_, NO_2,_ and CO showed the highest concentration during November–February (Nov–Feb) and the lowest during June–August (Jun–Aug). However, the monthly variation trend of O_3_-8h concentrations showed an opposite trend to the other five pollutants mentioned above, with a peak value appearing during Jun–Aug and the trough value during Nov–Feb. This observation was similar to previous studies [26]. It is not surprising that O_3_-8h has a different monthly variation trend than other pollutants, because a photochemical process produces O_3_-8h. Due to the prolong heating period (six months) within the HC during winter, the concentrations of the six air pollutants show a large variation between months of the year. For example, the monthly average of the PM_2.5_ concentration in the Harbin area reached 148 μg/m^3^ in November 2015, compared to only 27 μg/m^3^ in May 2015. In addition, the concentrations of the six air pollutants vary significantly in different cities within the same period. For instance, in January 2017, the PM_2.5_ and PM_10_ concentrations in Harbin were 123 and 148 μg/m^3^, respectively, while the concentrations of PM_2.5_ and PM_10_ in Yanbian during this period were only 54 and 66 μg/m^3^, respectively. In terms of monthly variation, the 11 cities within the HC region show a high spatial consistency among each other. The provincial capital cities have higher pollution levels than other cities. In addition, the monthly variation of PM_2.5_ showed an unobvious double peak during October–November (Oct–Nov) and March–April (Mar–Apr) during the OBB period. This result indicates that single pollutants are difficult to distinguish from OBB events. Therefore, the characteristic ratio of PM_10_/PM_2.5_ is used to distinguish OBB in the following sections.

### 3.2 Special pollution period

#### 3.2.1 The influence of dust weather

**Fig. 4:**
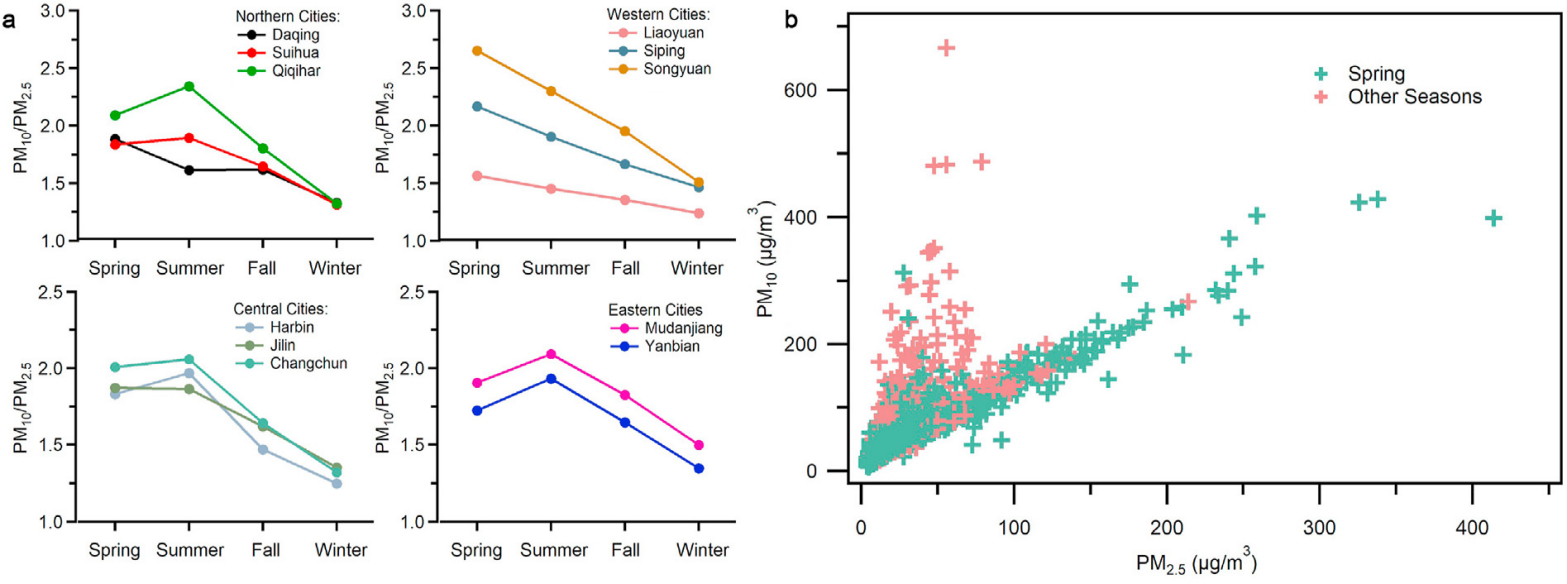
The influence of dust weather on HC region. **a**, The ratio of PM_10_/PM_2.5_in different seasons of 11 cities in HC. **b**, The scatter plot of PM_2.5_ and PM_10_ in Songyuan.

As mention above, the concentration of PM_10_ in Songyuan and Siping in spring was significantly higher than in other seasons. Therefore, we speculate that the reason may be that Songyuan and Siping are more vulnerable to sandstorms coming from Inner Mongolia in the spring. To prove this conjecture, the characteristic ratio of PM_10_ to PM_2.5_ is used to distinguish dust weather patterns according to the previous work [25]. The PM_10_/PM_2.5_ ratio within the different seasons of the HC is displayed in Fig. 4(a), and the daily variation is displayed in Fig. S7. The results show that the western cities all have the highest PM_10_/PM_2.5_ ratio in the spring. However, most northern, central, and western cities show a trend of the highest PM_10_/PM_2.5_ ratio during the summer. This result indicates that western cities suffered from stronger dusty weather than northern, central, and western cities in the spring. To confirm this view, a scatter plot of PM_2.5_ and PM_10_ pollution in Songyuan (Liaoyuan, Siping) is shown in Fig. 4(b) (Fig. S8(a), Fig. S8(b)). The scatter plot results show a linear correlation between PM_2.5_ and PM_10_ in the western cities within other seasons except for spring. The scatter plots of PM_2.5_ and PM_10_ pollution during the spring period further proves that the western cities are affected by sandstorm weather.

**Fig. 5:**
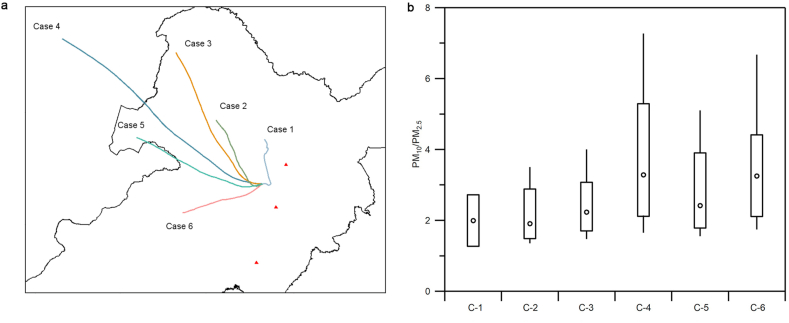
The backward trajectory cluster analysis during the spring period from 2015 to 2020 in Songyuan. **a**, The source direction of the six cases. **b**, The PM_10_/PM_2.5_ ratio in six cases.

In order to find the source of the spring dust, a back-trajectory cluster analysis during the spring period from 2015 to 2020 in Songyuan is shown in Fig. 5(a). According to the GIS-based software TrajStat, the 552 tracks were clustered into six cases. The proportions of the six cases are shown in Table. S3 and a box plot of PM_2.5_, PM_10_, SO_2_, NO_2_, CO, and O_3_-8h within the six cases are displayed in Fig. S9(a-f). As shown in Fig. 5(b), cases 4–6 through the Inner Mongolia region showed a higher PM_10_/PM_2.5_ ratio. Therefore, the back-trajectory cluster analyses helped us to prove that the dust from Inner Mongolia affected Songyuan. Furthermore, the dates with the highest PM_10_/PM_2.5_ ratios in the spring of that year were chosen for analysis in Fig. S10. The backward trajectories of all four days show air masses passing through the Inner Mongolia region and reaching the city of Songyuan. The scatter plot and the back-trajectory cluster analysis results helped us prove that the dust from Inner Mongolia affected the western cities Songyuan, Siping, and Liaoyuan during the spring period. In the geographical location, the western cities are more vulnerable to Inner Mongolian dust due to the cities being adjacent to Inner Mongolia. In general, the western cities Songyuan, Daqing, and Siping were more vulnerable to spring dust from Inner Mongolia according to the PM_10_/PM_2.5_ ratio and the back-trajectory cluster analysis.

#### 3.2.2 The influence of open biomass burning

**Fig. 6:**
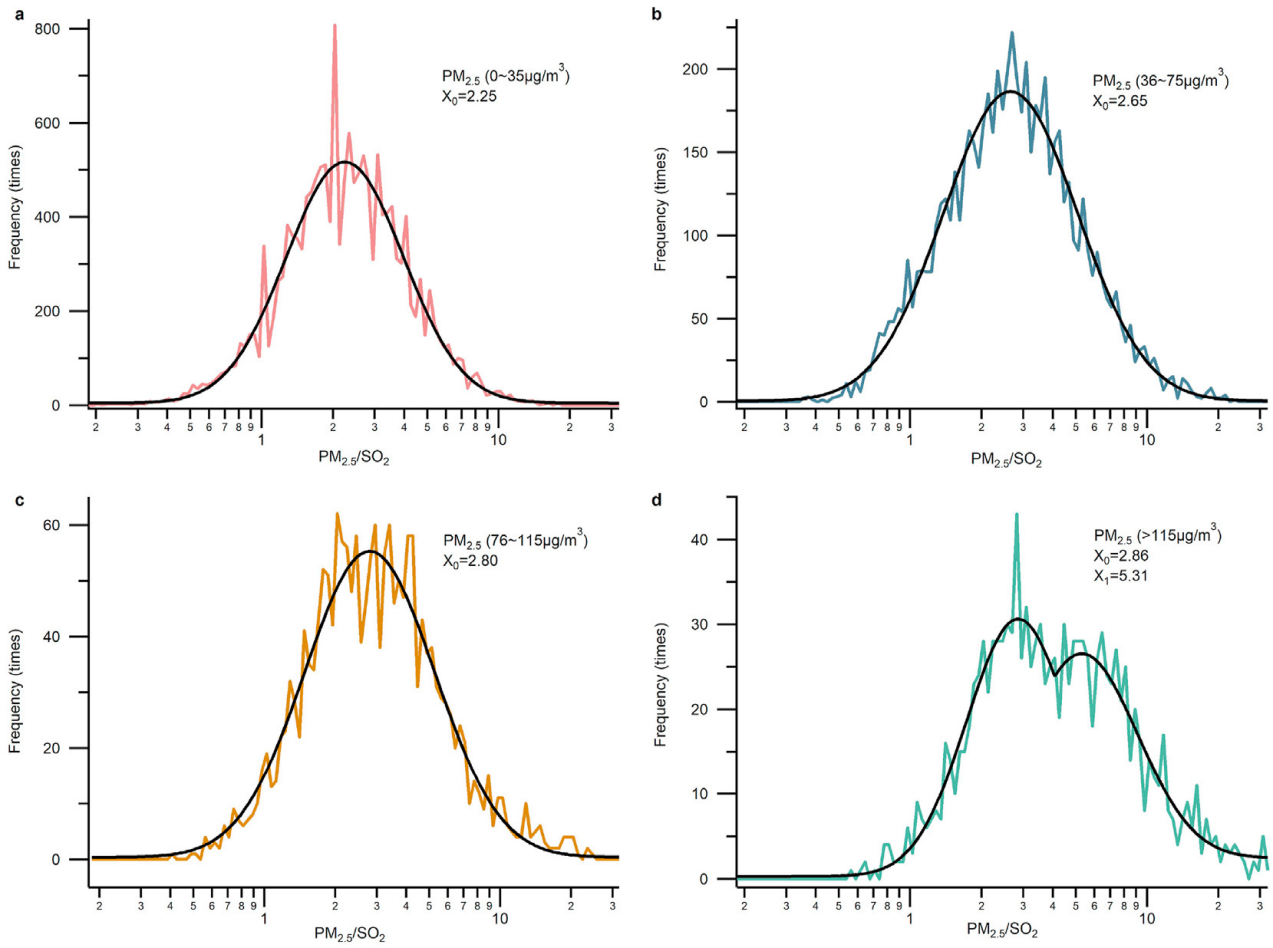
The frequency distribution of PM_2.5_/SO_2_ ratio at different PM_2.5_ concentrations. **a,** The frequency distribution of PM_2.5_/SO_2_ ratio when PM_2.5_ concentration is 0–35 μg/m^3^**b**, The frequency distribution of PM_2.5_/SO_2_ ratio when PM_2.5_ concentration is 36–75 μg/m^3^**c,** The frequency distribution of PM_2.5_/SO_2_ ratio when PM_2.5_ concentration is 76–115 μg/m^3^**d,** The frequency distribution of PM_2.5_/SO_2_ ratio when PM_2.5_ concentration is greater than 115 μg/m^3^.

OBB is a prevalent practice in the HC and has been reported by many studies. Cao et al [36] measured the emission factors of corn straw using a self-made combustion tower. The results showed that the emission factors of particulate matter and SO_2_ were 5.31 and 0.04 g/kg for corn straw and 6.28 and 0.18 g/kg for rice straw. Andreae et al [37] measured the emission factor of agricultural residues, and the results showed the emission factor of PM_2.5_ and SO_2_ is 3.9 and 0.4 g/kg, respectively. According to previous work, the particulate matter produced by biomass combustion is one order of magnitude higher than that of SO_2_. In the observation of the HC area, it was found that when the number of fire spots in the map is large, the ratio of PM_2.5_/SO_2_ is also higher. This phenomenon can be reflected in the corresponding time period shown in Fig. S14 and Fig. S11. Therefore, it is beneficial to use the ratio of PM_2.5_ to SO_2_ to identify OBB. The distribution frequency of PM_2.5_/SO_2_ with a step of 10∧(0.02) at different PM_2.5_ concentrations were analyzed in this work in order to measure the effects of the PM_2.5_/SO_2_ ratio, and is shown in Fig. 6(a–d). The frequency distribution results showed that when the PM_2.5_ concentration was higher than 115 μg/m^3^, the bimodal distribution replaced the log-normal distribution. The frequency distribution provides further evidence that it is beneficial to use the ratio of PM_2.5_/SO_2_ in order to identify OBB, especially during the high PM_2.5_ concentration period.

The ratio of PM_2.5_/SO_2_ was also used to identify biomass combustion events within this work and is displayed in Fig. S11. As shown in Fig. S11(a), the high ratio of PM_2.5_/SO_2_ frequently appeared from June to August in the summer, which may be caused by the low concentration of SO_2_. Fig. S11(b, c, d) showed the ratio of PM_2.5_/SO_2_ without the data of SO_2_ below 6, 7, and 8 μg/m^3^. The comparison of the graphs shows that the reason for the high PM_2.5_/SO_2_ ratio during summer is due to the low concentration of SO_2_. Moreover, the differences in Fig. S11(b), Fig. S11(c), and Fig. S11(d) are not apparent, the Fig. S12 showed the PM_2.5_/SO_2_ in each city of HC (the data with SO_2_ concentration lower than 6 μg/m^3^ are excluded). Furthermore, the daily variation of the mass concentrations of PM_2.5_ and SO_2_ in the 11 cities is shown in Fig. S13. According to air quality data, the concentration of SO_2_ is often below 5 μg/m^3^ during the summer period, in which case the PM_2.5_/SO_2_ ratio will be high and not suitable for identifying OBB. Therefore, removing the SO_2_ concentration data below 6 μg/m^3^ in the analysis of the PM_2.5_/SO_2_ ratio is scientifically meaningful in order to identify the OBB phenomenon.

The daily variation of PM_2.5_ displayed in Fig. S13 indicates that most of the high PM_2.5_ values occur during the heating period. In addition, the daily trend of SO_2_ concentration is similar to that of PM_2.5_. According to Fig. S11(b-d), the peak PM_2.5_/SO_2_ value appeared primarily during Mar–Apr and Oct–Nov, in which the OBB frequently occurred during this time. From 2014 to 2017, the ratio of PM_2.5_/SO_2_ occurred more frequently during Oct–Nov than in Mar–Apr, implying that farmers are more inclined to burn straw during the fall. The fire points diagram was used to confirm this idea further and is shown in Fig. S14. In 2018, the peak PM_2.5_/SO_2_ ratio value showed a bimodal distribution during Mar–Apr and Oct–Nov which was influence primarily by the government's burning prohibition. From 2019 to 2020, the ratio of PM_2.5_/SO_2_ occurred more frequently during Mar–Apr than in Oct–Nov. The peak PM_2.5_/SO_2_ ratio shifted from Oct–Nov to Mar–Apr from 2014 to 2020, which was affected by the burning prohibition. From September 15, 2019 to May 15, 2020, Heilongjiang provincial government implemented a regional ban on OBB; however, many fire points appeared in April 2020 according to Fig. S14. The PM_2.5_/SO_2_ ratio and the fire points map show that the burning prohibition did not work during this period. The OBB events that occurred in early April 2020 indicated that the burning prohibition only delayed air pollution and did not fundamentally improve the air quality. In summary, OBB frequently occurred during Mar–Apr and Oct–Nov, and has been confirmed by the ratio of PM_2.5_/SO_2_. Furthermore, OBB shifted from Oct–Nov to Mar–Apr during 2014–2020, and the PM_2.5_/SO_2_ ratio and the fire points were observed.

## 4 Conclusions

The concentrations of PM_2.5_, PM_10_, SO_2_, NO_2,_ and CO showed a similar trend in annual variation (decrease from 2014 to 2017 and stable from 2018 to 2020) in most cities except Suihua. The decrease of SO_2_ was the most apparent among the six pollutants related to the government measures used to reduce emissions, such as eliminating small coal-fired boilers. The seasonal variation of the PM_2.5_, SO_2_, NO_2,_ and CO concentrations were the lowest in the summer and highest in the winter. The monthly variations of the concentrations of PM_2.5_, SO_2_, NO_2,_ and CO show a similar trend, with the highest value during Nov–Feb and the lowest value during Jun–Aug. However, the concentrations of O_3_-8h showed an opposite trend to the other five pollutants, with the highest value during Jun–Aug and the lowest value during Nov–Feb.

The scatter plots of PM_2.5_ and PM_10_ proved that the western cities Songyuan, Daqing, and Siping were more vulnerable to spring dust storms. The back-trajectory cluster analyses confirmed that the source of the dust was coming from Inner Mongolia. Moreover, OBB is very common in HC, showing a bimodal distribution during Oct–Nov, and Mar–Apr. The OBB moved from Oct–Nov to Mar–Apr due to the burning prohibition from 2014 to 2020. The burning prohibition only delayed air pollution and did not fundamentally improve the air quality.

## Credit author statement

Yulong Wang: Investigation, Writing - Original Draft. Yuan Cheng: Conceptualization, Methodology, Writing - Review & Editing. Zhiqing Zhang: Validation. Youwen Sun: Writing - Review & Editing.

## Declaration of competing interest

The authors declare that they have no known competing financial interests or personal relationships that could have appeared to influence the work reported in this paper.
