# Supplementary material for: Spatiotemporal variation and source analysis of air pollutants in the Harbin-Changchun (HC) region of China during 2014–2020
Source: Environ Sci Ecotechnol. 2021 Sep 15;8:100126. doi: 10.1016/j.ese.2021.100126 (PMC9488001; doi:10.1016/j.ese.2021.100126)
Supplement: Multimedia component 1 [file mmc1.docx]

**Supporting information for:**

# Spatiotemporal variation and source analysis of air pollutants in the Harbin-Changchun (HC) region of China during 2014-2020

Yulong Wang ^1^, Youwen Sun ^2,*^, Zhiqing Zhang ^1^, Yuan Cheng ^1,*^

*^1^ State Key Laboratory of Urban Water Resource and Environment, School of Environment, Harbin Institute of Technology, Harbin 150090, China*

*^2^ Key Laboratory of Environmental Optics and Technology, Anhui Institute of Optics and Fine Mechanics, Chinese Academy of Sciences, Hefei 230031, China*

**Corresponding author. Youwen Sun; E-mail address: ywsun@aiofm.ac.cn*

*Yuan Cheng; E-mail address: ycheng@hit.edu.cn*

**SI-1 The monitoring technology of target pollutants**

According to China Environmental Protection Standards HJ 655-2013 (<http://www.mee.gov.cn/ywgz/fgbz/bz/bzwb/jcffbz/201308/W020130802492823718666.pdf>), the concentrations of PM_2.5_ and PM_10_ (the particulate matter with the aerodynamic equivalent diameter of less than 2.5 and 10µm, respectively) are measured by the micro oscillating balance method and the β absorption method, respectively. According to China Environmental Protection Standards HJ 193-2013 (<http://www.mee.gov.cn/ywgz/fgbz/bz/bzwb/jcffbz/201308/W020130802493970989627.pdf>), the ultraviolet fluorescence method, chemiluminescence method and UV-spectrophotometry method are used to measure the mass concentration of SO_2_, NO_2_ and O_3,_ respectively. Besides, the gas filter correlation infrared absorption method and the non-dispersive infrared absorption method are used to measure the CO concentration. According to the acceptance requirements of HJ 655-2013 and HJ 193-2013, the measurement error of PM_2.5_, PM_10_, SO_2_, NO_2_, O_3_ and CO is ±2%, ±2%, ±2%, ±2%, ±4% and ±2%, respectively.

**
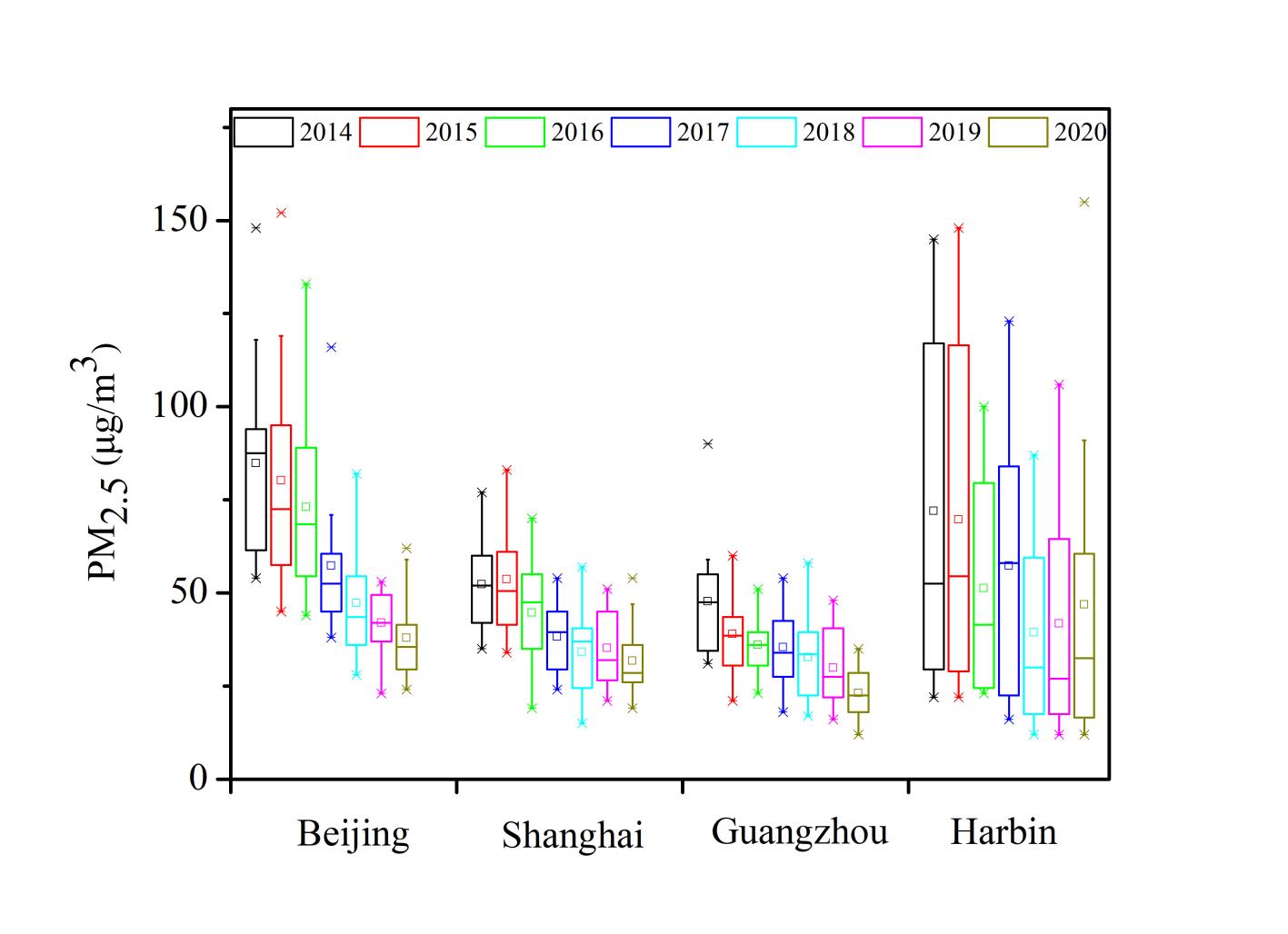
**

**Fig. S1.** Comparison of annual variation of PM_2.5_ in Beijing, Shanghai, Guangzhou and Harbin.

**
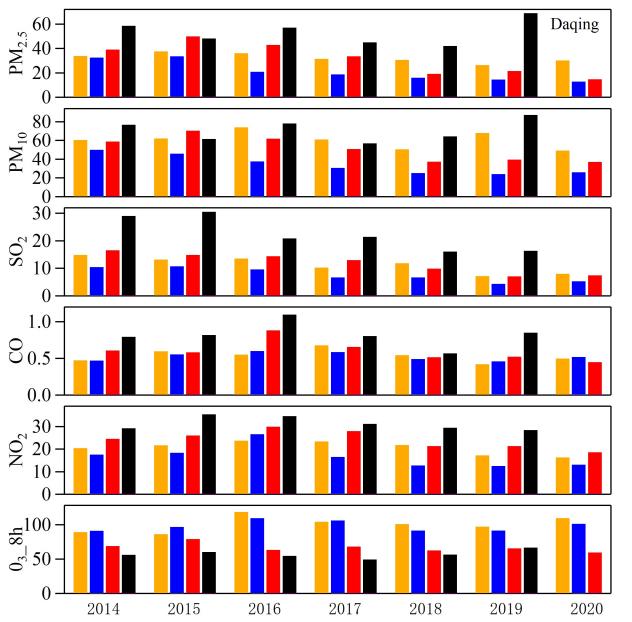

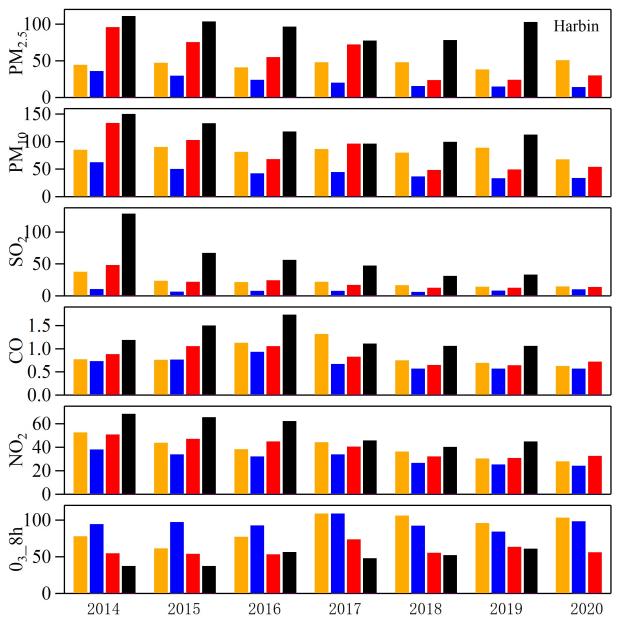

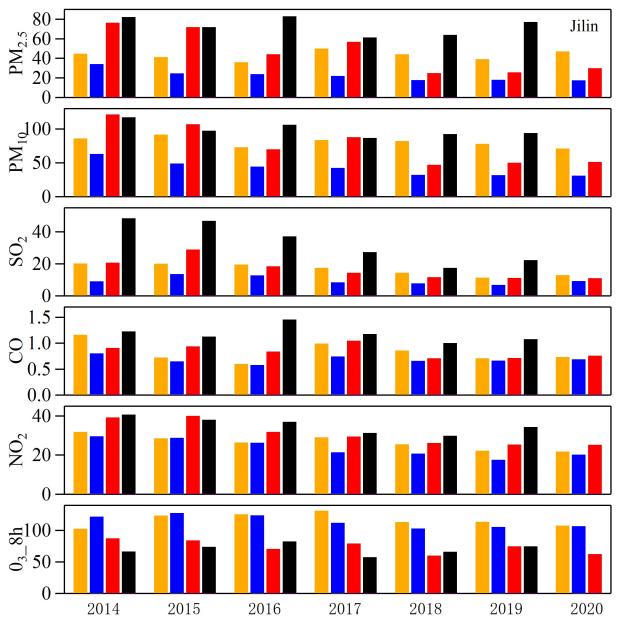

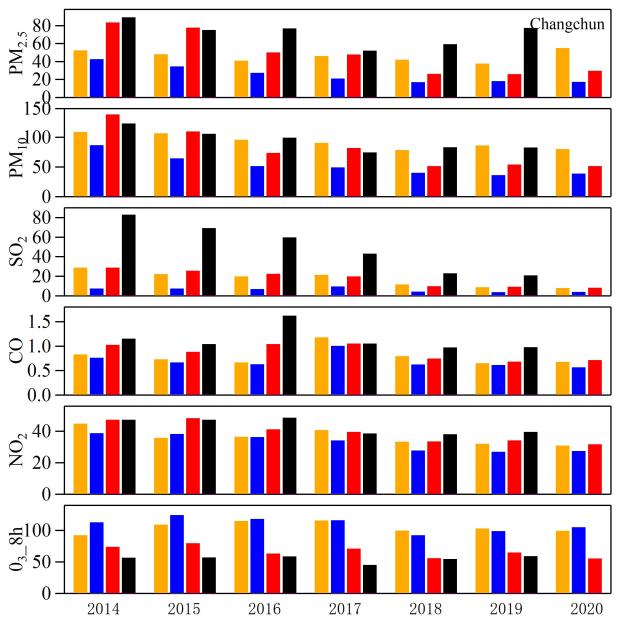
**

**
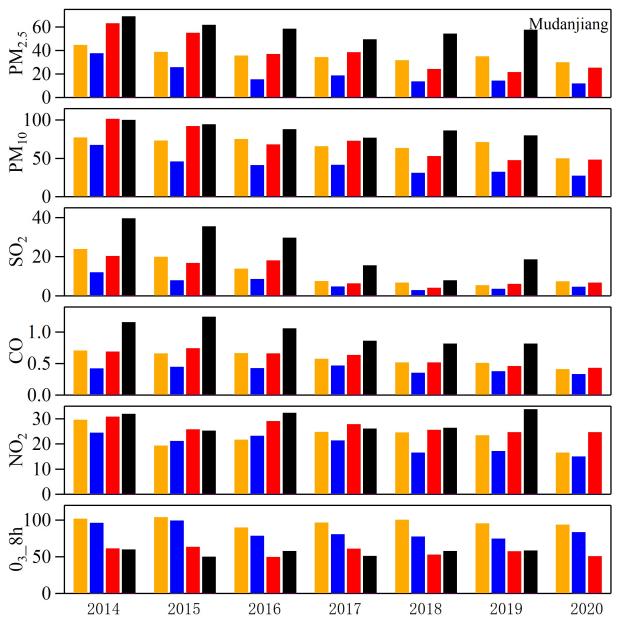

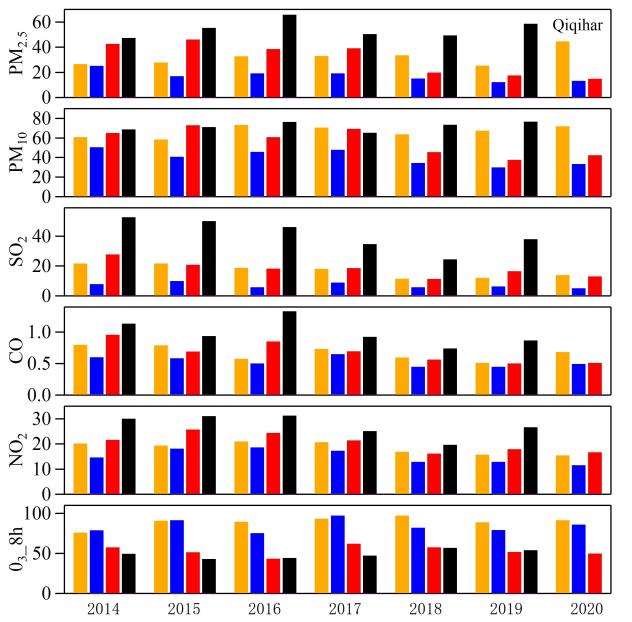

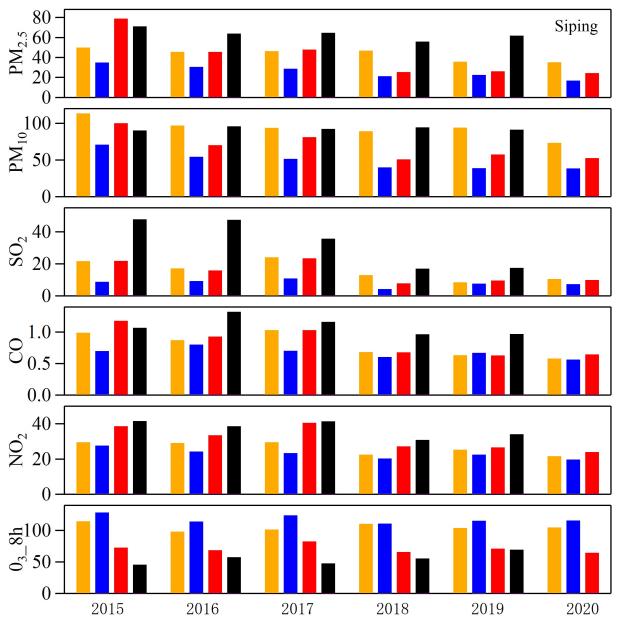

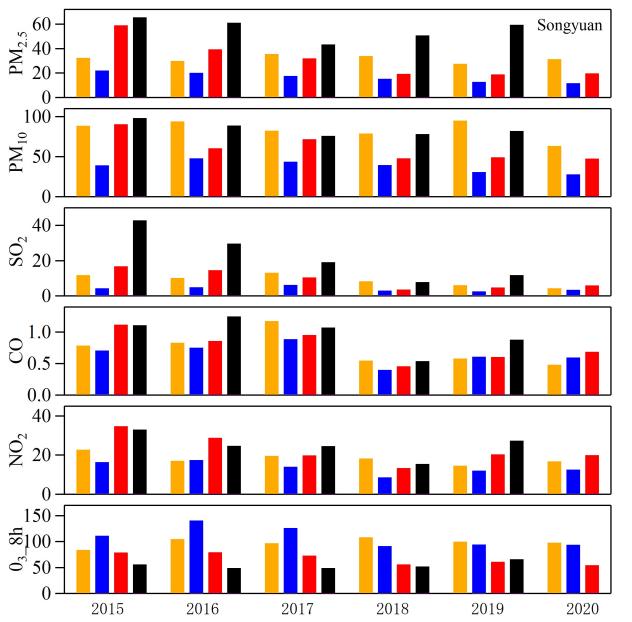

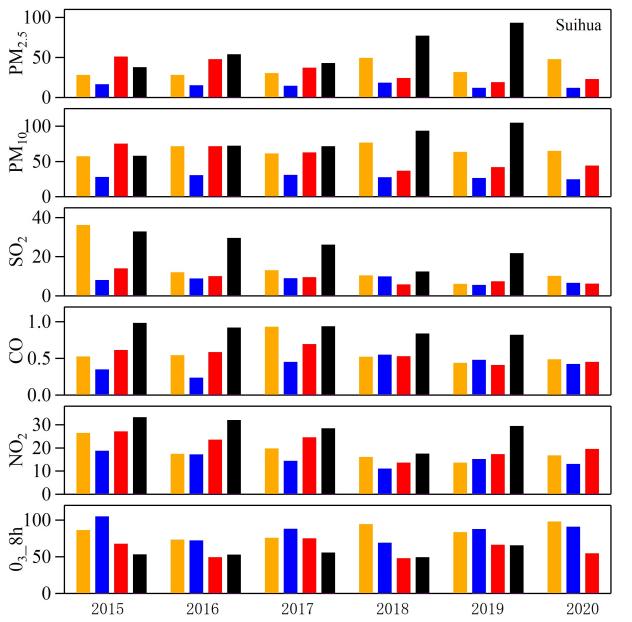

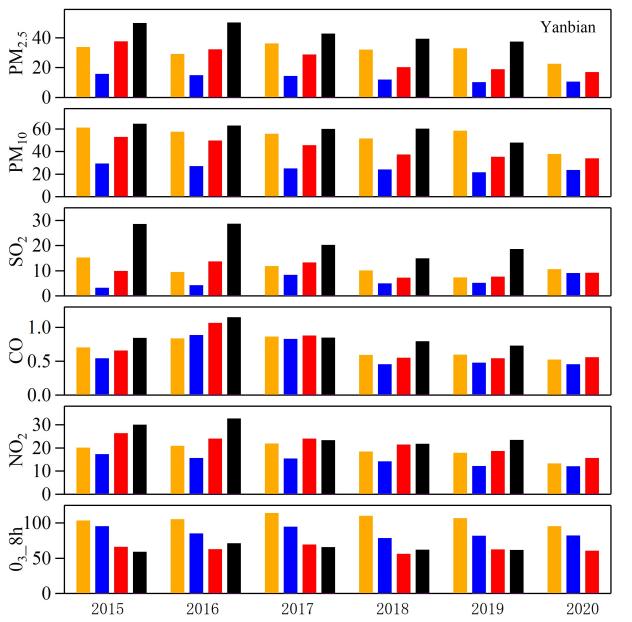

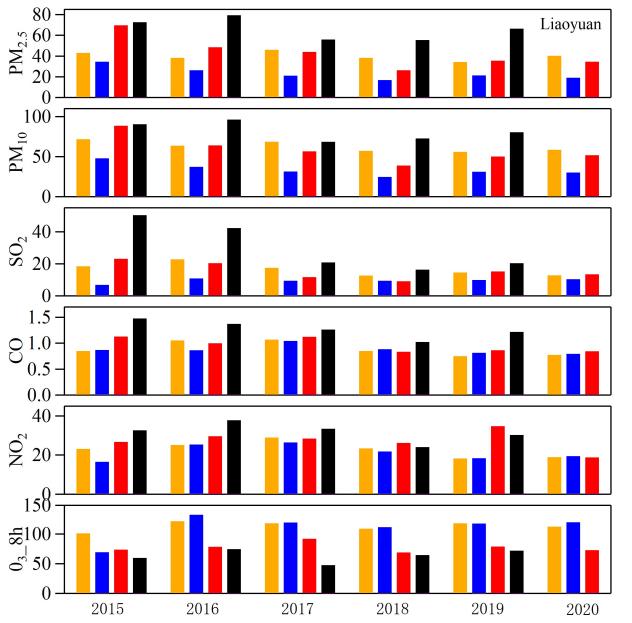
**

**Fig. S2.** The interannual variation of the seasonal average mass concentrations of six criteria pollutants in 11 cities of HC during 2014–2020. The units of mass concentrations are μg/m^3^ for PM_2.5_, PM_10_, SO_2_, NO_2_, O_3__8h, and mg/m^3^ for CO. The four colors of yellow, blue, red and black represent the four seasons of spring, summer, autumn and winter, respectively.


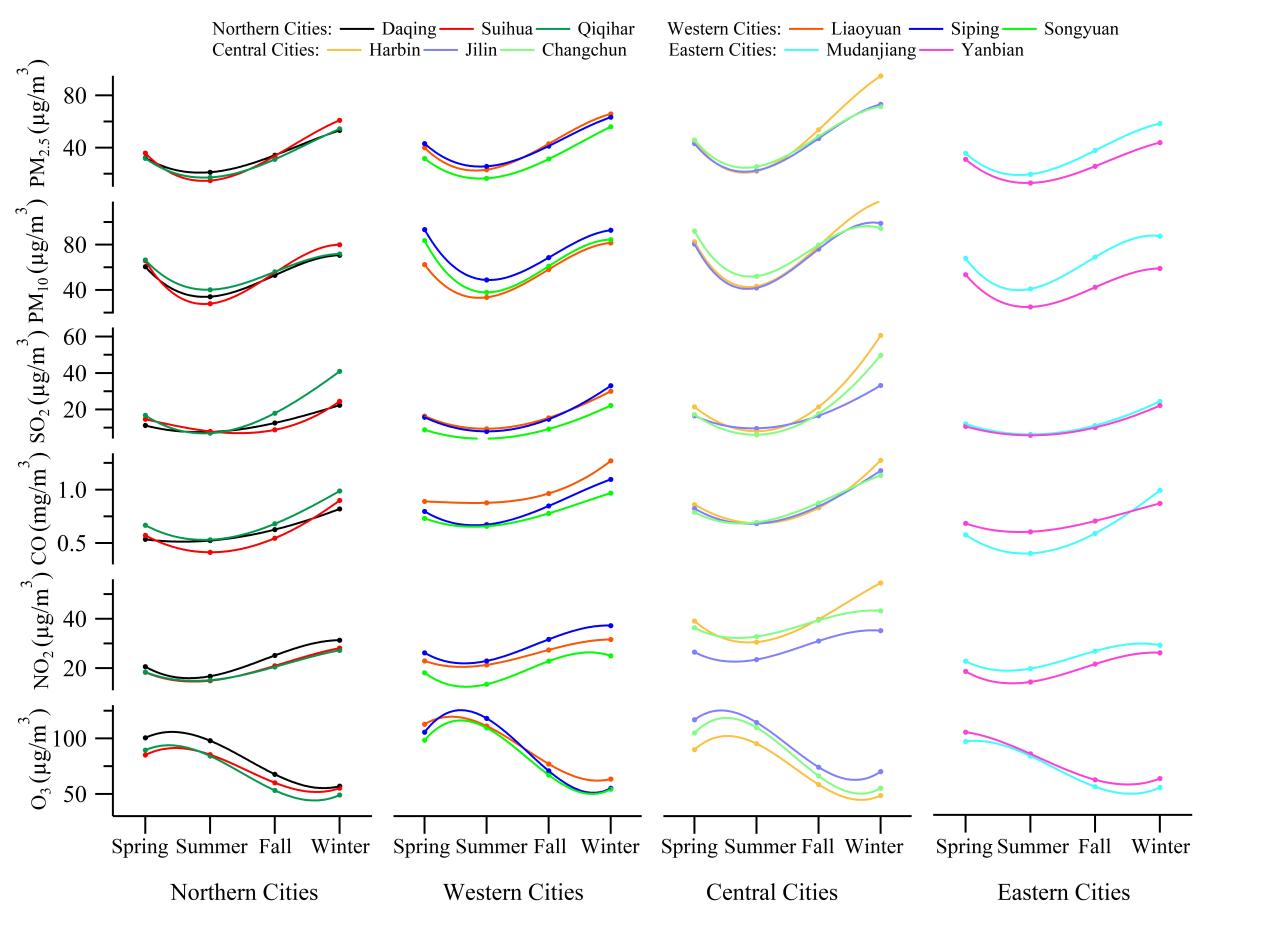


**Fig. S3.** The seasonal trend analysis of six criteria pollutants in 11 cities of HC during 2014–2020. The units of mass concentrations are μg/m^3^ for PM_2.5_, PM_10_, SO_2_, NO_2_, O_3_, and mg/m^3^ for CO.


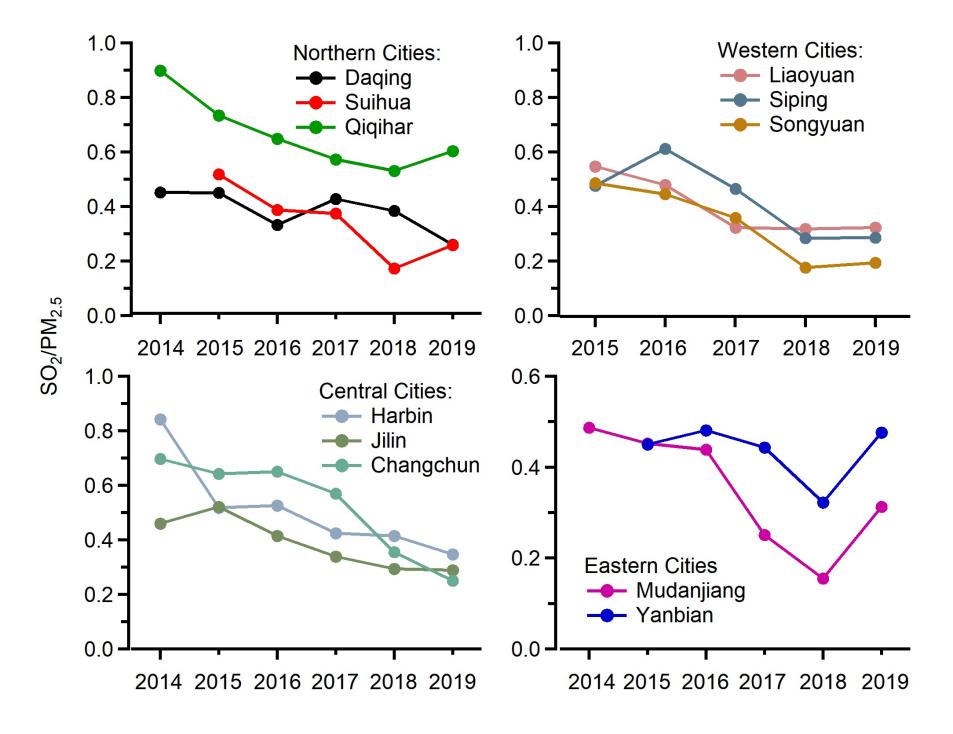


## Fig. S4. The interannual variation of the SO_2_/PM_2.5_ during the heating period.

## **Fig. S5**. The interannual variation of NO_2_/SO_2_ ratio during the heating period in 11 cities of HC.

##
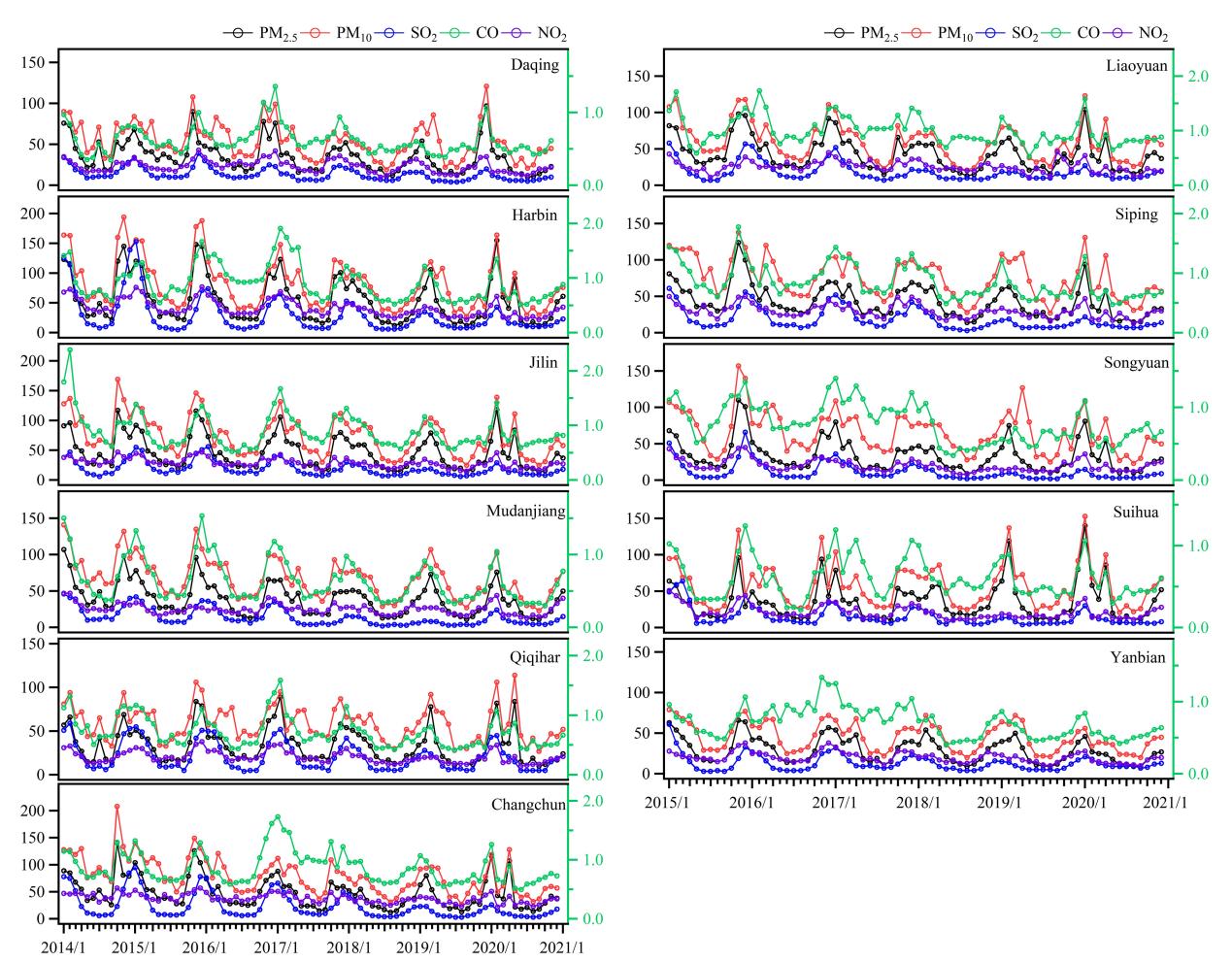


**Fig. S6.** The monthly average mass concentrations of PM_2.5_, PM_10_, SO_2_, CO and NO_2_ in 11 cities of HC. The units of mass concentrations are μg/m^3^ for PM_2.5_, PM_10_, SO_2_, NO_2_, and mg/m^3^ for CO.


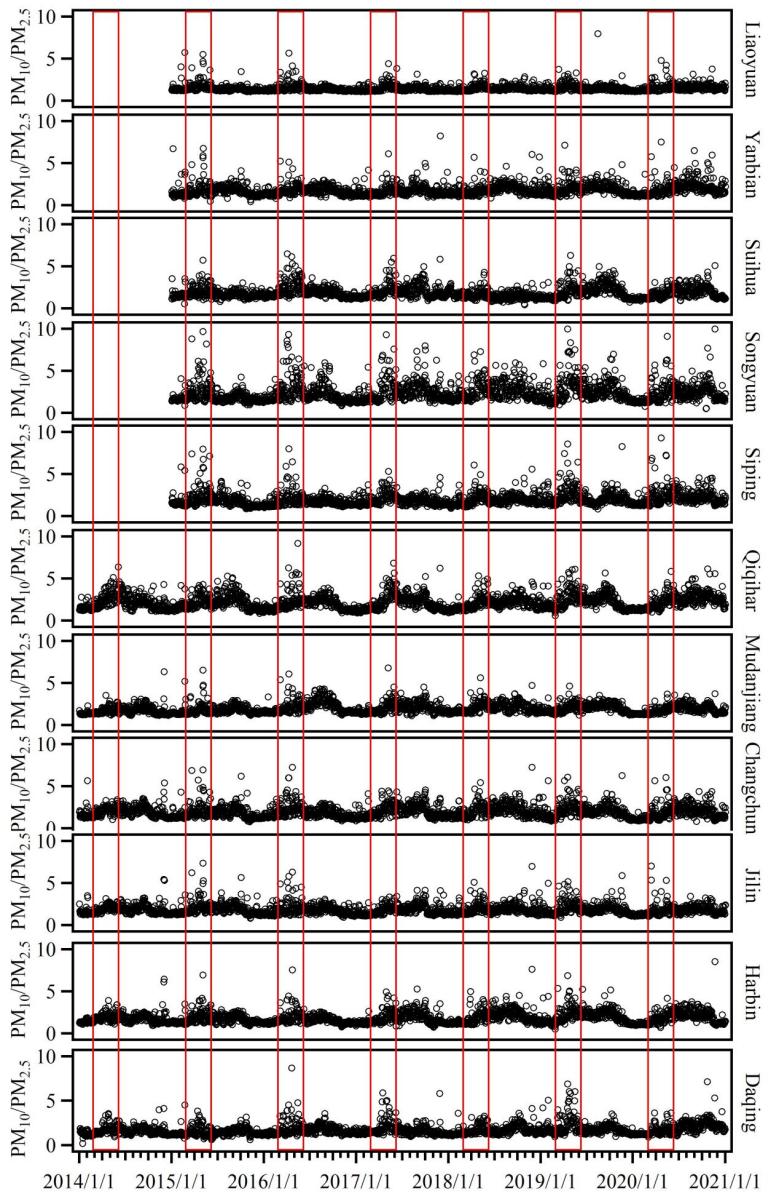


## Fig. S7. The ratio of daily PM_10_/PM_2.5_ in 11 cities of HC during 2014-2020. The red part represent the spring.


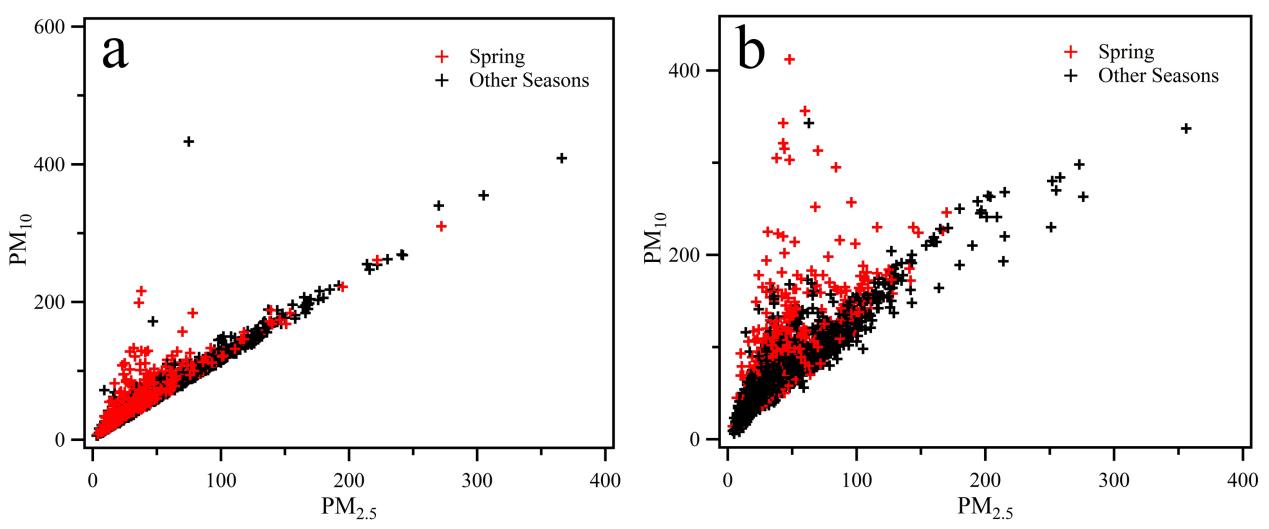


## Fig. S8. The scatter plot of PM_2.5_ and PM_10_ in Liaoyuan(a) and Siping(b).


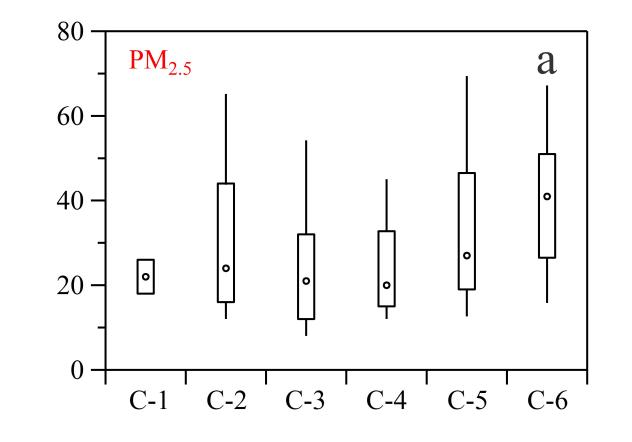

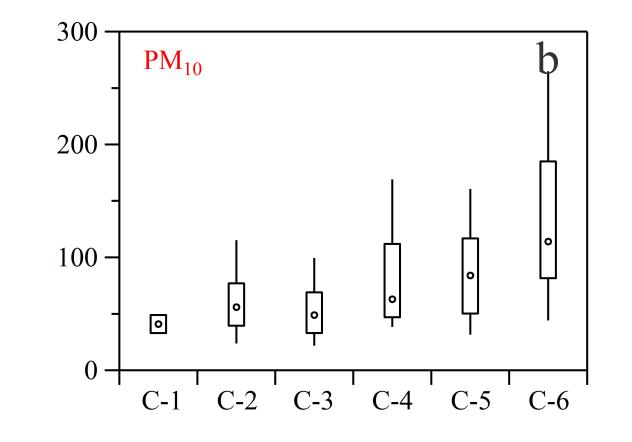

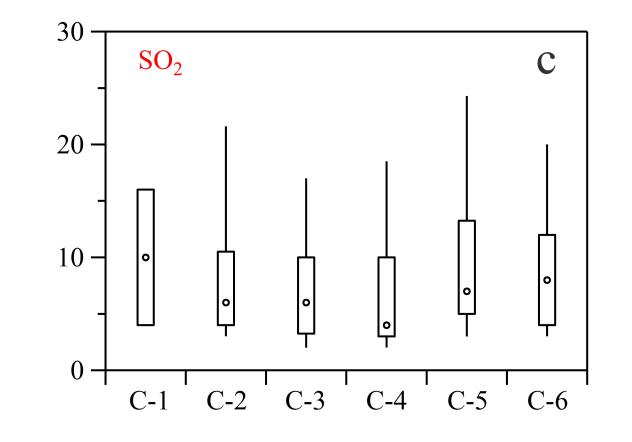

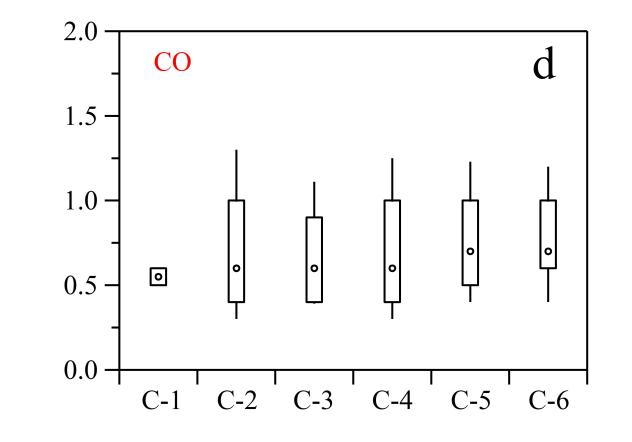

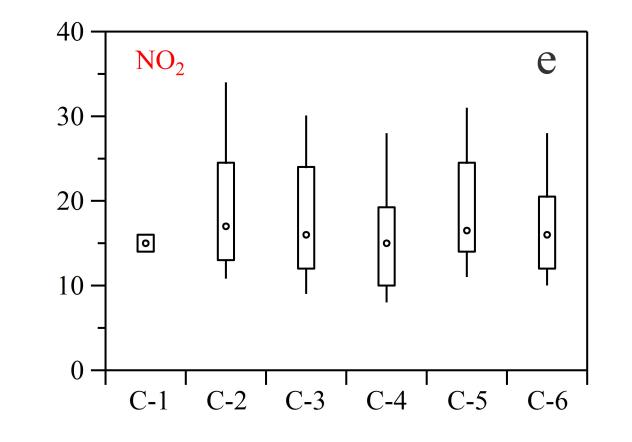

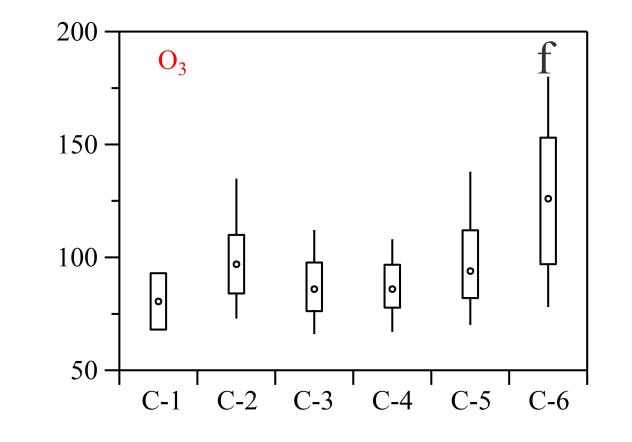


## Fig. S9. The box plot of PM_2.5_, PM_10_, SO_2_, NO_2_, CO and O_3_-8h in six cases were displayed in Fig. S5(a-f), respectively.


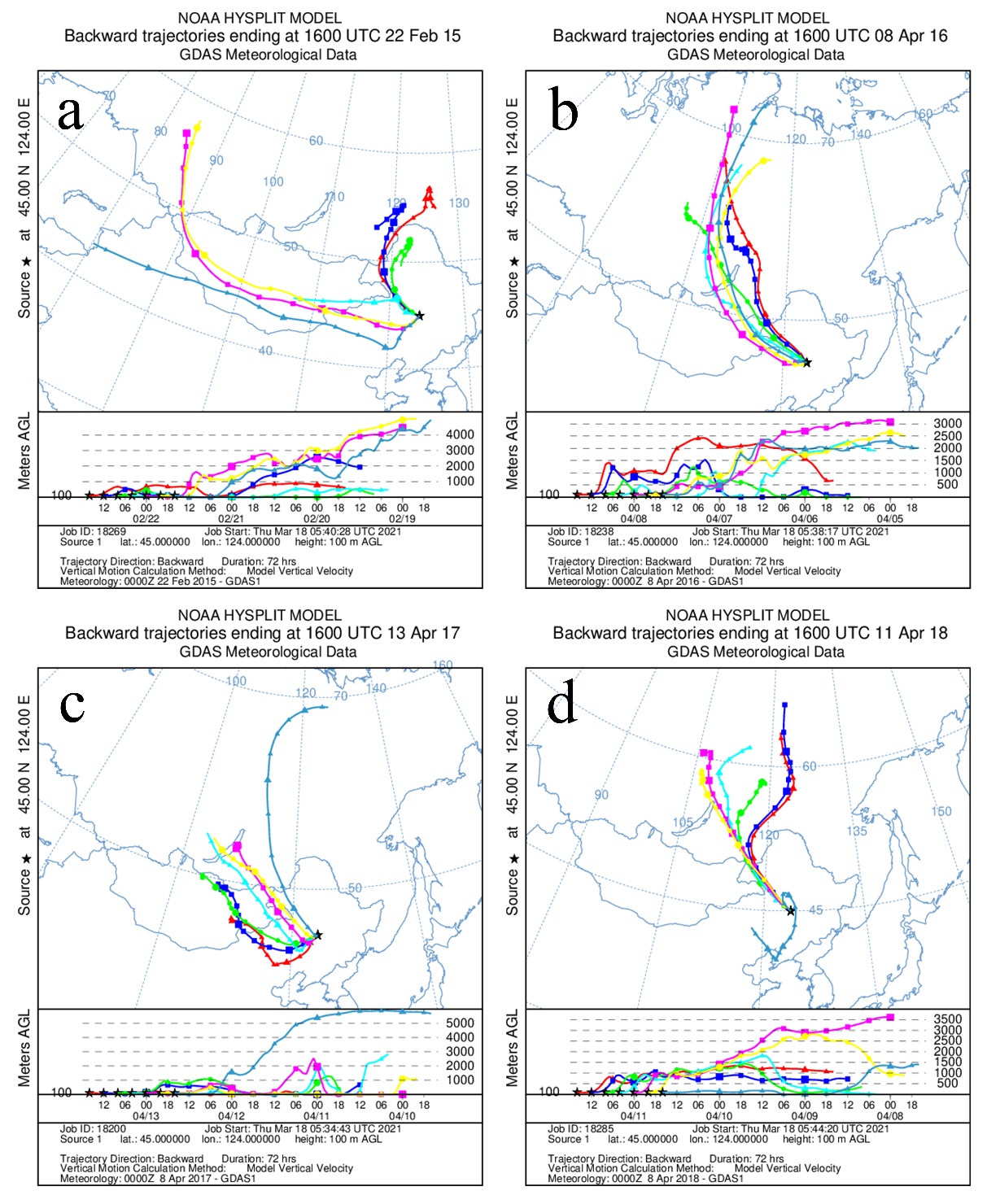


## Fig. S10. The backward trajectory analysis of the day with the highest PM_10_/PM_2.5_ ratio in Songyuan of HC (a) 2015.02.22 (b) 2016.04.08 (c) 2017.04.13 (d) 2018.04.11.


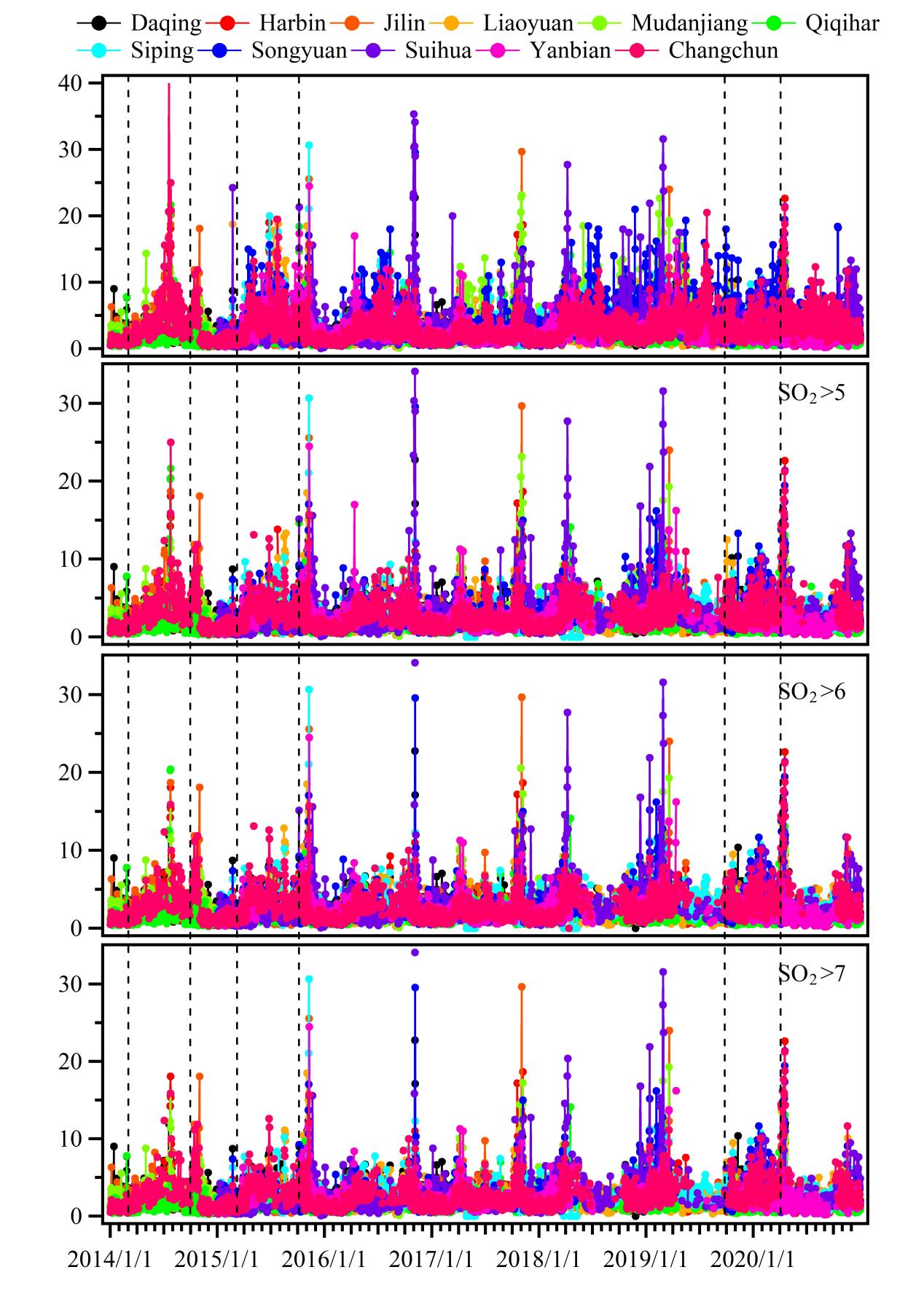


## Fig. S11. The ratio of PM_2.5_/SO_2_ in 11 cities of HC (a) with all SO_2_ data (b) without the data of SO_2_ below 6μg/m^3^ (c) without the data of SO_2_ below 7μg/m^3^ (d) without the data of SO_2_ below 8μg/m^3^.


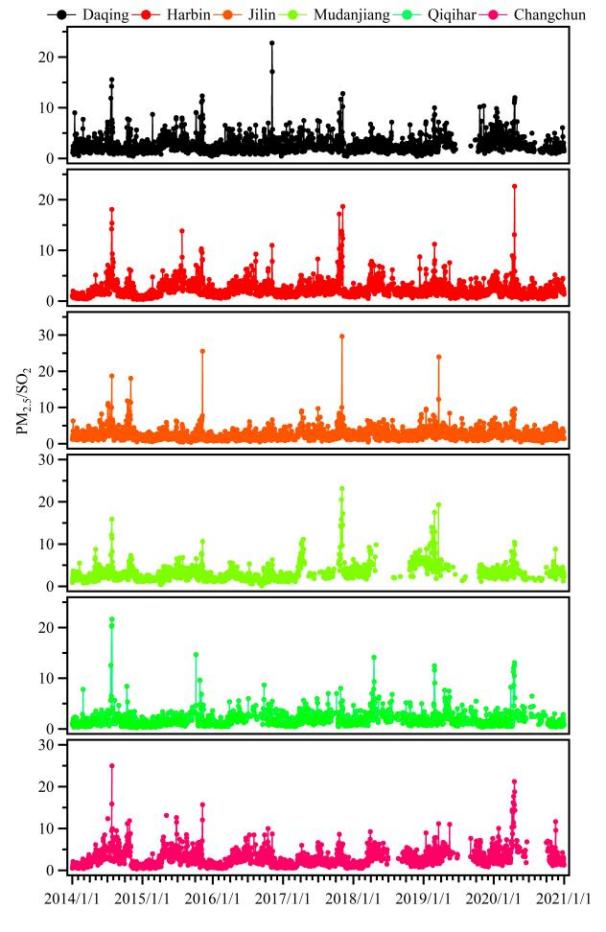

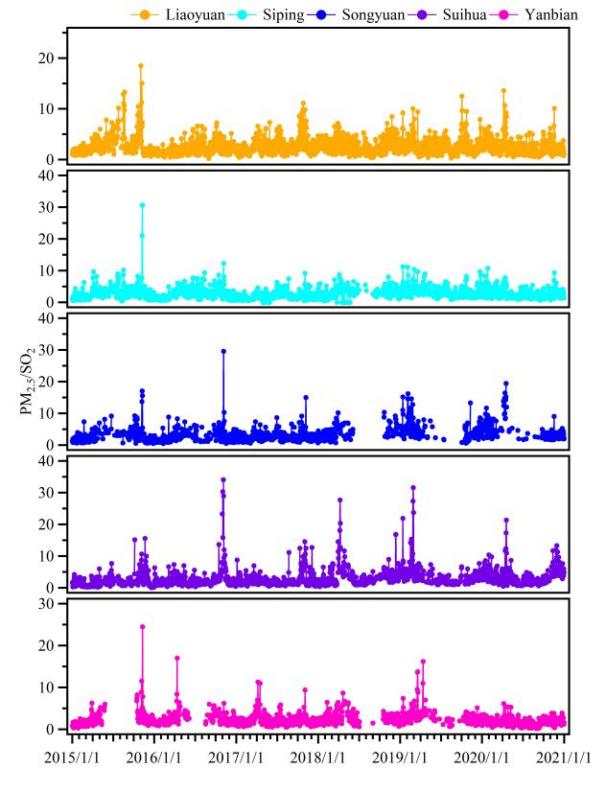


## Fig. S12. The data of PM_2.5_/SO_2_ without the data of SO_2_ below 6 μg/m^3^ in 11 cities of HC.


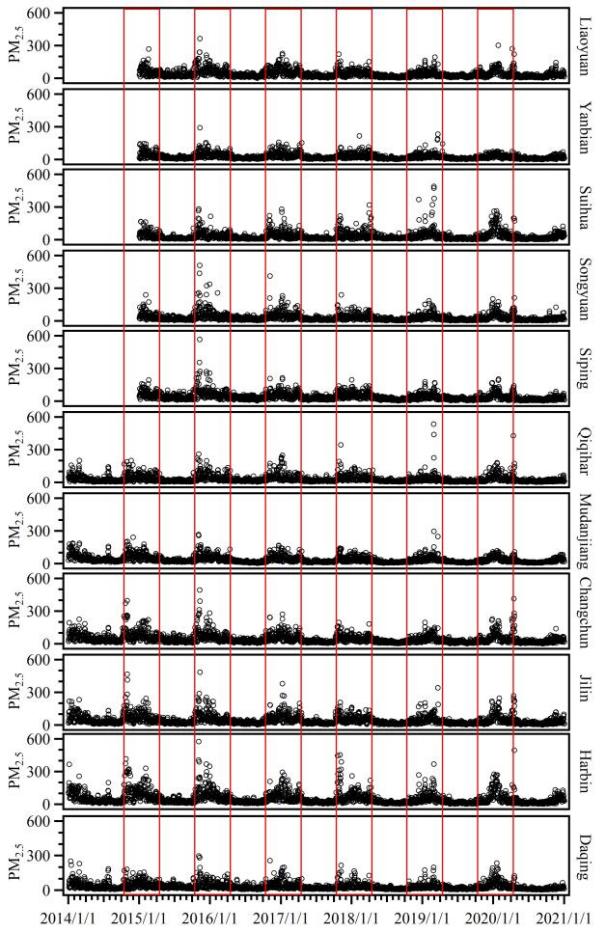

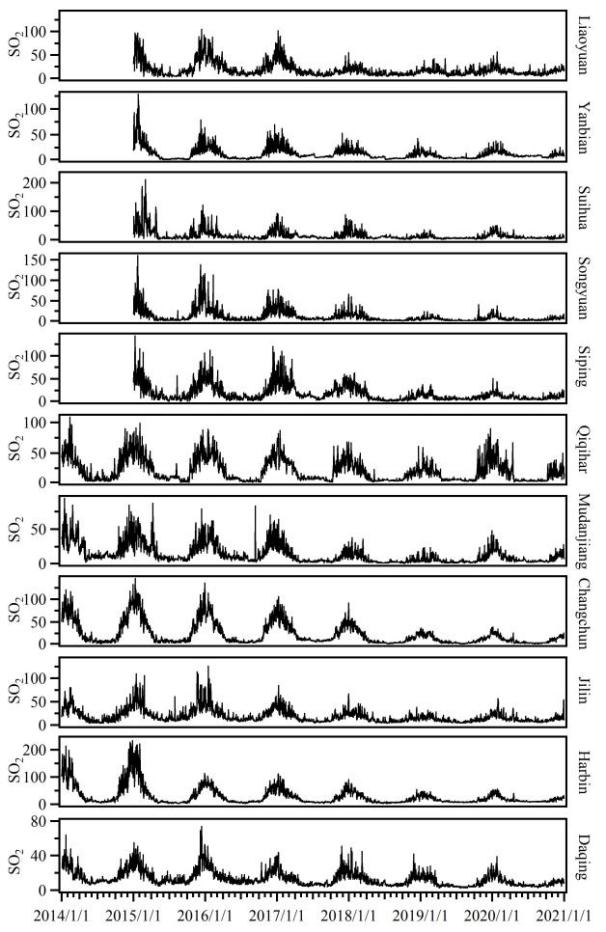


## Fig. S13. The daily variation of the mass concentrations of PM_2.5_ and SO_2_ in 11 cities of HC. The units of mass concentrations are μg/m^3^.


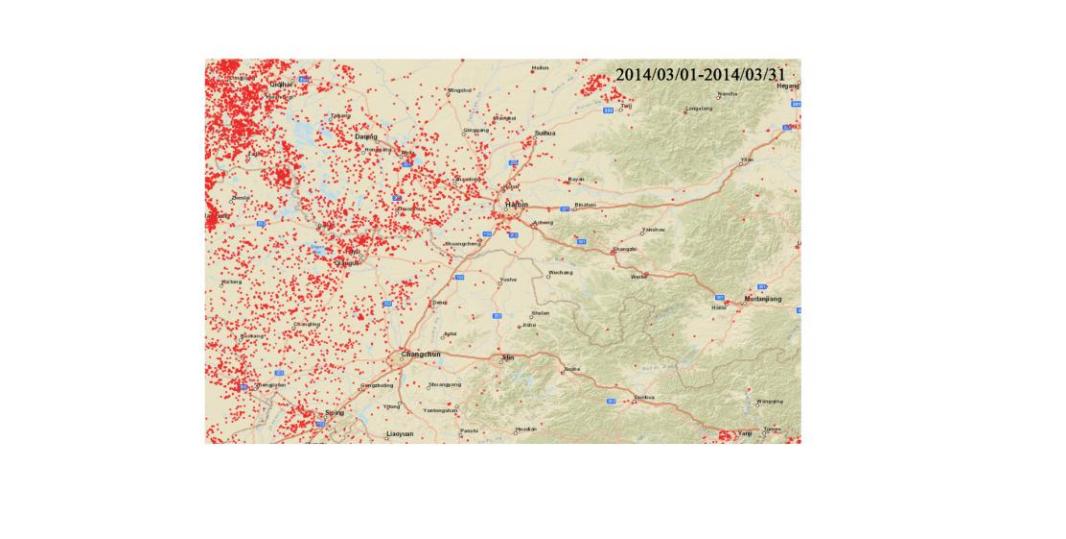

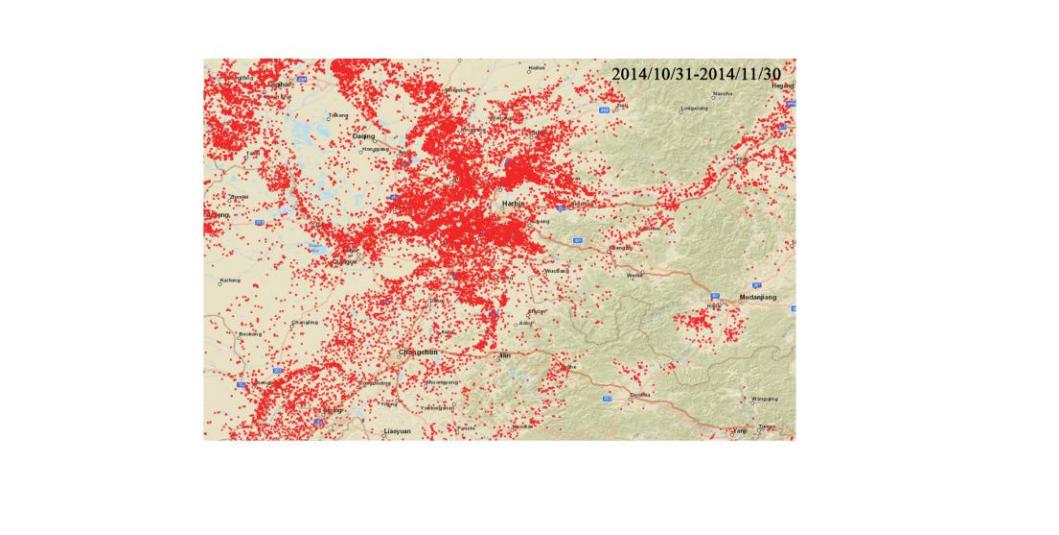


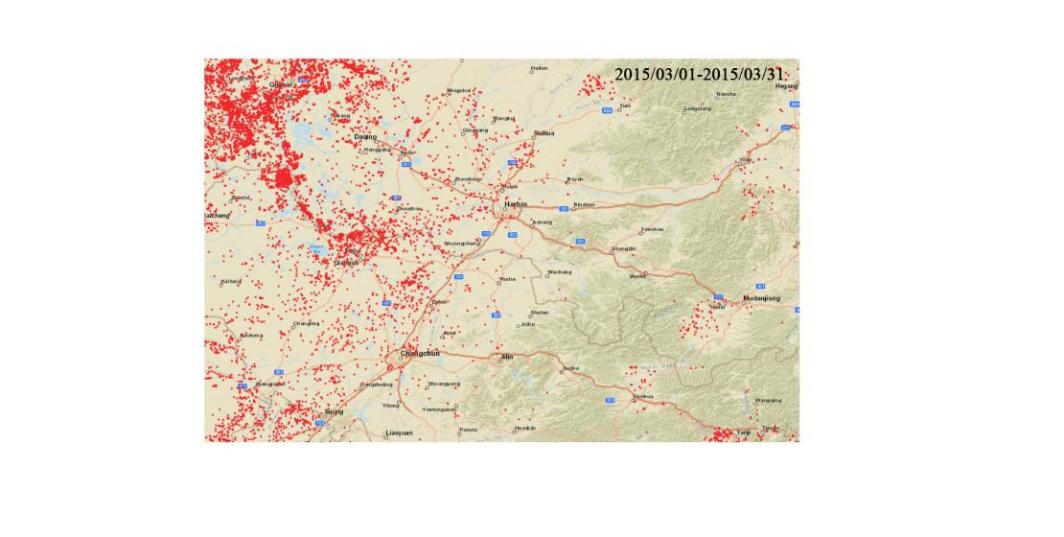

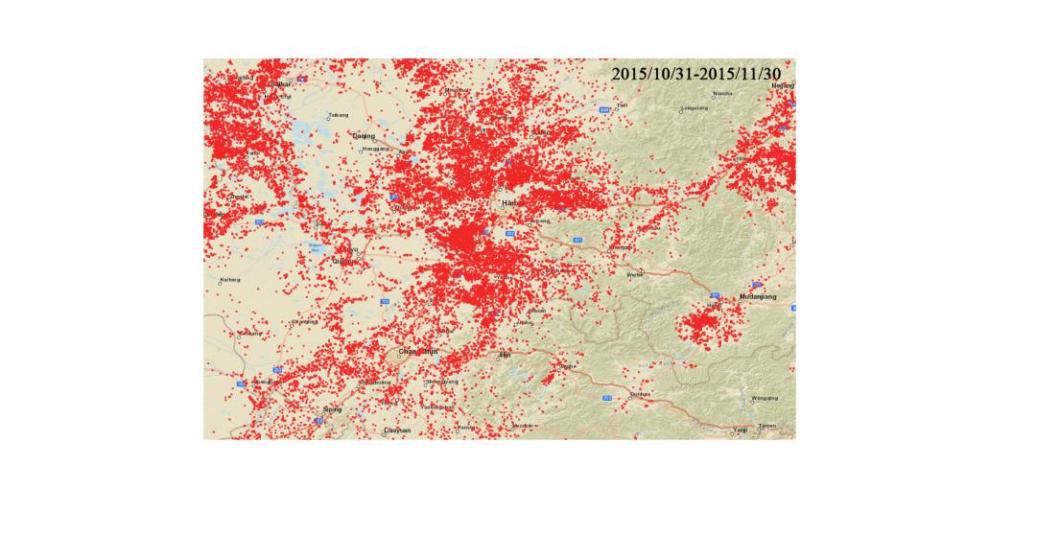


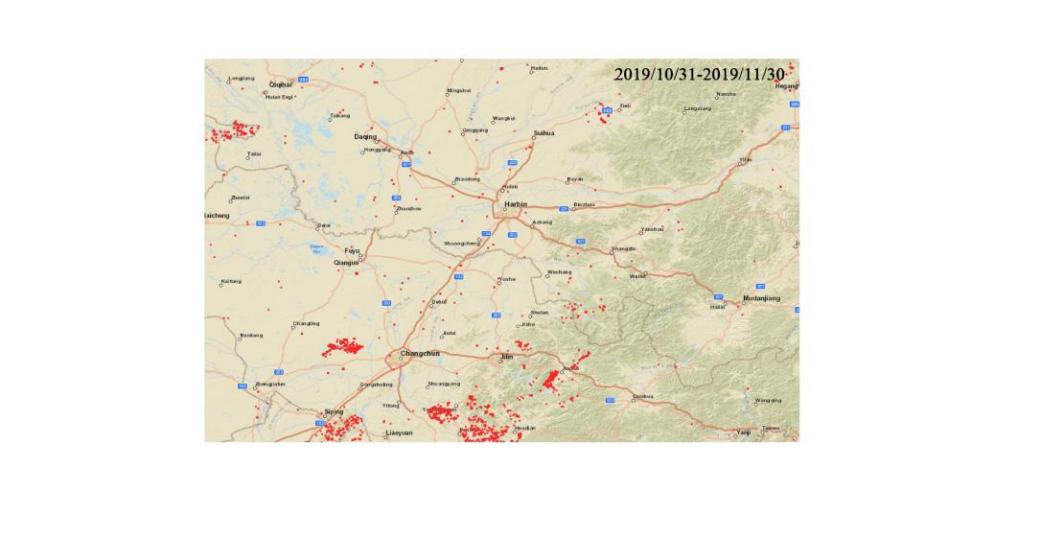

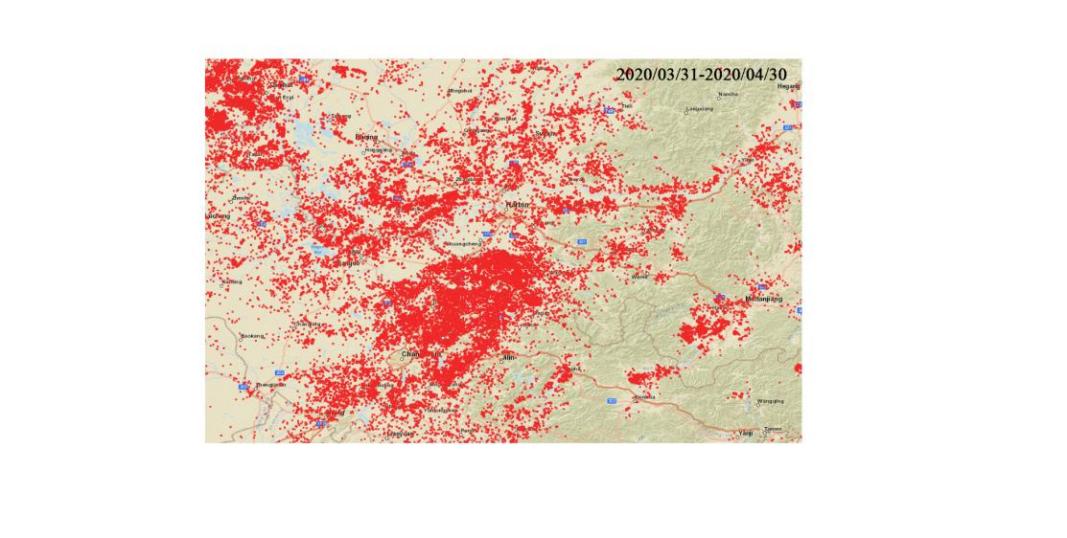


## Fig. S14. The spatial distributions of fire points during six periods. Period 1 (2014.03.01-2014.03.31), Period 2 (2014.10.31-2014.11.31), Period 3 (2015.03.01-2015.03.31), Period 4 (2015.10.31-2015.11.30), Period 5 (2019.10.31-2019.11.30), Period 6 (2020.03.31-2020.04.30).

**Table S1.** The annual average mass concentrations of six pollutants in 11cities during 2014 to 2020. The units of mass concentrations are μg/m^3^ for PM_2.5_, PM_10_, SO_2_, NO_2_, O_3_-8h, and mg/m^3^ for CO.

| Daqing |  | PM_2.5_ | PM_10_ | SO_2_ | CO | NO_2_ | O_3_-8h |
| --- | --- | --- | --- | --- | --- | --- | --- |
|  | 2014 | 42.98 | 62.80 | 17.72 | 0.60 | 23.07 | 75.16 |
|  | 2015 | 44.42 | 63.12 | 17.80 | 0.64 | 25.16 | 79.91 |
|  | 2016 | 37.21 | 59.79 | 15.75 | 0.71 | 28.21 | 87.29 |
|  | 2017 | 33.94 | 52.54 | 12.56 | 0.74 | 25.58 | 81.68 |
|  | 2018 | 26.41 | 41.81 | 11.73 | 0.55 | 21.08 | 77.73 |
|  | 2019 | 28.70 | 51.74 | 8.62 | 0.51 | 20.38 | 77.11 |
|  | 2020 | 27.93 | 46.38 | 8.69 | 0.56 | 18.08 | 84.26 |
| Harbin |  | PM_2.5_ | PM_10_ | SO_2_ | CO | NO_2_ | O_3_-8h |
|  | 2014 | 71.55 | 108.63 | 55.43 | 0.92 | 52.09 | 66.54 |
|  | 2015 | 69.33 | 101.98 | 38.90 | 0.99 | 49.58 | 62.58 |
|  | 2016 | 51.14 | 74.70 | 28.89 | 1.14 | 43.73 | 65.90 |
|  | 2017 | 57.57 | 84.44 | 25.15 | 1.11 | 44.19 | 86.21 |
|  | 2018 | 39.42 | 63.68 | 18.50 | 0.74 | 34.03 | 76.80 |
|  | 2019 | 41.41 | 69.95 | 16.52 | 0.75 | 32.50 | 73.32 |
|  | 2020 | 46.78 | 64.58 | 17.42 | 0.73 | 32.02 | 80.44 |
| Jilin |  | PM_2.5_ | PM_10_ | SO_2_ | CO | NO_2_ | O_3_-8h |
|  | 2014 | 60.09 | 98.05 | 22.85 | 1.15 | 34.66 | 93.18 |
|  | 2015 | 57.09 | 93.26 | 28.37 | 0.91 | 35.36 | 101.24 |
|  | 2016 | 41.67 | 68.26 | 23.39 | 0.79 | 29.76 | 98.76 |
|  | 2017 | 52.05 | 79.06 | 18.37 | 1.05 | 29.18 | 99.30 |
|  | 2018 | 35.42 | 60.21 | 13.93 | 0.81 | 24.94 | 85.43 |
|  | 2019 | 37.61 | 63.11 | 11.87 | 0.78 | 24.37 | 90.40 |
|  | 2020 | 40.41 | 59.58 | 13.77 | 0.80 | 24.72 | 87.87 |
| Liaoyuan |  | PM_2.5_ | PM_10_ | SO_2_ | CO | NO_2_ | O_3_-8h |
|  | 2014 | 58.03 | 80.46 | 25.24 | 1.08 | 26.08 | 76.64 |
|  | 2015 | 45.91 | 63.02 | 24.60 | 1.10 | 28.58 | 99.04 |
|  | 2016 | 44.10 | 59.02 | 18.50 | 1.15 | 29.58 | 97.81 |
|  | 2017 | 33.15 | 46.42 | 12.18 | 0.90 | 24.98 | 86.70 |
|  | 2018 | 37.25 | 52.53 | 14.31 | 0.89 | 24.49 | 94.44 |
|  | 2019 | 38.62 | 54.40 | 14.27 | 0.88 | 20.92 | 93.87 |
|  | 2020 | 58.03 | 80.46 | 25.24 | 1.08 | 26.08 | 76.64 |
| Mudanjiang |  | PM_2.5_ | PM_10_ | SO_2_ | CO | NO_2_ | O_3_-8h |
|  | 2014 | 57.70 | 91.16 | 24.57 | 0.77 | 31.51 | 78.68 |
|  | 2015 | 47.51 | 78.72 | 20.67 | 0.79 | 24.15 | 80.50 |
|  | 2016 | 36.58 | 68.87 | 18.79 | 0.72 | 25.78 | 67.70 |
|  | 2017 | 36.09 | 64.88 | 10.39 | 0.67 | 25.57 | 73.41 |
|  | 2018 | 29.03 | 55.44 | 6.43 | 0.54 | 22.94 | 71.62 |
|  | 2019 | 32.49 | 60.36 | 6.77 | 0.55 | 23.94 | 70.84 |
|  | 2020 | 30.58 | 50.83 | 9.01 | 0.49 | 22.63 | 72.13 |
| Qiqihar |  | PM_2.5_ | PM_10_ | SO_2_ | CO | NO_2_ | O_3_-8h |
|  | 2014 | 37.39 | 63.48 | 27.66 | 0.88 | 21.70 | 61.58 |
|  | 2015 | 37.16 | 63.02 | 25.90 | 0.80 | 24.02 | 70.21 |
|  | 2016 | 35.26 | 61.28 | 22.74 | 0.74 | 23.32 | 62.90 |
|  | 2017 | 37.88 | 64.55 | 22.28 | 0.83 | 22.27 | 74.19 |
|  | 2018 | 27.80 | 51.46 | 13.93 | 0.59 | 16.65 | 72.76 |
|  | 2019 | 27.76 | 52.99 | 16.52 | 0.56 | 17.58 | 67.96 |
|  | 2020 | 29.98 | 54.16 | 15.56 | 0.62 | 16.58 | 70.87 |
| Siping |  | PM_2.5_ | PM_10_ | SO_2_ | CO | NO_2_ | O_3_-8h |
|  | 2015 | 61.79 | 100.27 | 26.78 | 1.06 | 35.45 | 91.40 |
|  | 2016 | 45.49 | 76.63 | 21.71 | 0.91 | 31.48 | 82.95 |
|  | 2017 | 46.35 | 79.86 | 25.87 | 1.02 | 33.28 | 90.01 |
|  | 2018 | 37.06 | 65.48 | 12.72 | 0.73 | 26.00 | 85.66 |
|  | 2019 | 35.65 | 71.66 | 10.68 | 0.73 | 26.81 | 86.69 |
|  | 2020 | 32.62 | 61.41 | 11.08 | 0.67 | 24.26 | 89.51 |
| Songyuan |  | PM_2.5_ | PM_10_ | SO_2_ | CO | NO_2_ | O_3_-8h |
|  | 2015 | 47.37 | 83.31 | 20.71 | 0.96 | 28.31 | 80.57 |
|  | 2016 | 35.08 | 70.21 | 14.81 | 0.88 | 22.49 | 94.96 |
|  | 2017 | 35.14 | 70.61 | 14.33 | 1.06 | 19.71 | 85.33 |
|  | 2018 | 26.90 | 58.79 | 6.92 | 0.56 | 15.12 | 76.88 |
|  | 2019 | 29.44 | 65.31 | 5.77 | 0.62 | 16.66 | 77.10 |
|  | 2020 | 27.81 | 51.85 | 6.00 | 0.64 | 18.70 | 78.16 |
| Suihua |  | PM_2.5_ | PM_10_ | SO_2_ | CO | NO_2_ | O_3_-8h |
|  | 2015 | 36.18 | 59.31 | 27.12 | 0.64 | 29.66 | 79.34 |
|  | 2016 | 33.22 | 58.81 | 15.08 | 0.55 | 22.07 | 61.49 |
|  | 2017 | 34.34 | 58.25 | 14.68 | 0.76 | 22.36 | 72.75 |
|  | 2018 | 33.99 | 51.68 | 11.48 | 0.61 | 15.69 | 67.72 |
|  | 2019 | 37.03 | 58.42 | 8.64 | 0.54 | 17.39 | 71.27 |
|  | 2020 | 41.57 | 57.37 | 10.22 | 0.53 | 19.26 | 77.31 |
| Yanbian |  | PM_2.5_ | PM_10_ | SO_2_ | CO | NO_2_ | O_3_-8h |
|  | 2015 | 36.72 | 54.96 | 17.97 | 0.71 | 23.50 | 80.05 |
|  | 2016 | 30.90 | 49.16 | 13.49 | 0.92 | 22.53 | 79.44 |
|  | 2017 | 30.74 | 45.76 | 14.95 | 0.91 | 22.47 | 86.26 |
|  | 2018 | 26.30 | 43.01 | 10.01 | 0.59 | 18.95 | 78.05 |
|  | 2019 | 25.50 | 43.44 | 8.74 | 0.60 | 17.76 | 78.30 |
|  | 2020 | 20.87 | 35.08 | 11.47 | 0.55 | 15.51 | 75.58 |
| Changchun |  | PM_2.5_ | PM_10_ | SO_2_ | CO | NO_2_ | O_3_-8h |
|  | 2014 | 65.46 | 113.23 | 35.87 | 0.93 | 44.10 | 83.43 |
|  | 2015 | 64.24 | 102.55 | 33.79 | 0.88 | 43.52 | 92.70 |
|  | 2016 | 46.22 | 78.71 | 28.15 | 0.87 | 39.78 | 88.20 |
|  | 2017 | 45.99 | 78.34 | 26.38 | 1.18 | 40.17 | 89.31 |
|  | 2018 | 31.98 | 58.61 | 14.63 | 0.77 | 32.43 | 75.22 |
|  | 2019 | 37.85 | 65.76 | 10.92 | 0.74 | 33.60 | 80.38 |
|  | 2020 | 42.00 | 61.29 | 9.91 | 0.71 | 31.71 | 79.91 |

**Table S2.** The Pearson Correlation in different month in 11 cities of HC.

| Daqing |  | PM_2.5_ | PM_10_ | SO_2_ | CO | NO_2_ | O_3_-8h |
| --- | --- | --- | --- | --- | --- | --- | --- |
|  | PM_2.5_ | 1 | 0.913** | 0.794** | 0.781** | 0.764** | -0.441** |
|  | PM_10_ | 0.913** | 1 | 0.679** | 0.642** | 0.693** | -0.301** |
|  | SO_2_ | 0.794** | 0.679** | 1 | 0.733** | 0.821** | -0.573** |
|  | CO | 0.781** | 0.642** | 0.733** | 1 | 0.818** | -0.521** |
|  | NO_2_ | 0.764** | 0.693** | 0.821** | 0.818** | 1 | -0.579** |
|  | O_3_-8h | -0.441** | -0.301** | -0.573** | -0.521** | -0.579** | 1 |
| Harbin |  | PM_2.5_ | PM_10_ | SO_2_ | CO | NO_2_ | O_3_-8h |
|  | PM_2.5_ | 1 | 0.957** | 0.745** | 0.756** | 0.842** | -0.548** |
|  | PM_10_ | 0.957** | 1 | 0.776** | 0.726** | 0.869** | -0.508** |
|  | SO_2_ | 0.745** | 0.776** | 1 | 0.647** | 0.837** | -0.591** |
|  | CO | 0.756** | 0.726** | 0.647** | 1 | 0.822** | -0.450** |
|  | NO_2_ | 0.842** | 0.869** | 0.837** | 0.822** | 1 | -0.575** |
|  | O_3_-8h | -0.548** | -0.508** | -0.591** | -0.450** | -0.575** | 1 |
| Jilin |  | PM_2.5_ | PM_10_ | SO_2_ | CO | NO_2_ | O_3_-8h |
|  | PM_2.5_ | 1 | 0.955** | 0.780** | 0.801** | 0.836** | -0.380** |
|  | PM_10_ | 0.955** | 1 | 0.742** | 0.748** | 0.844** | -0.270* |
|  | SO_2_ | 0.780** | 0.742** | 1 | 0.761** | 0.771** | -0.414** |
|  | CO | 0.801** | 0.748** | 0.761** | 1 | 0.691** | -0.443** |
|  | NO_2_ | 0.836** | 0.844** | 0.771** | 0.691** | 1 | -0.328** |
|  | O_3_-8h | -0.380** | -0.270* | -0.414** | -0.443** | -0.328** | 1 |
| Liaoyuan |  | PM_2.5_ | PM_10_ | SO_2_ | CO | NO_2_ | O_3_-8h |
|  | PM_2.5_ | 1 | 0.975** | 0.783** | 0.758** | 0.642** | -0.519** |
|  | PM_10_ | 0.975** | 1 | 0.780** | 0.713** | 0.610** | -0.457** |
|  | SO_2_ | 0.783** | 0.780** | 1 | 0.742** | 0.587** | -0.428** |
|  | CO | 0.758** | 0.713** | 0.742** | 1 | 0.621** | -0.445** |
|  | NO_2_ | 0.642** | 0.610** | 0.587** | 0.621** | 1 | -0.262* |
|  | O_3_-8h | -0.519** | -0.457** | -0.428** | -0.445** | -0.262* | 1 |
| Mudanjiang |  | PM_2.5_ | PM_10_ | SO_2_ | CO | NO_2_ | O_3_-8h |
|  | PM_2.5_ | 1 | 0.968** | 0.820** | 0.899** | 0.776** | -0.346** |
|  | PM_10_ | 0.968** | 1 | 0.783** | 0.866** | 0.774** | -0.287** |
|  | SO_2_ | 0.820** | 0.783** | 1 | 0.884** | 0.661** | -0.320** |
|  | CO | 0.899** | 0.866** | 0.884** | 1 | 0.741** | -0.468** |
|  | NO_2_ | 0.776** | 0.774** | 0.661** | 0.741** | 1 | -0.448** |
|  | O_3_-8h | -0.346** | -0.287** | -0.320** | -0.468** | -0.448** | 1 |
| Qiqihar |  | PM_2.5_ | PM_10_ | SO_2_ | CO | NO_2_ | O_3_-8h |
|  | PM_2.5_ | 1 | 0.895** | 0.791** | 0.823** | 0.826** | -0.454** |
|  | PM_10_ | 0.895** | 1 | 0.647** | 0.688** | 0.718** | -0.244* |
|  | SO_2_ | 0.791** | 0.647** | 1 | 0.868** | 0.886** | -0.636** |
|  | CO | 0.823** | 0.688** | 0.868** | 1 | 0.847** | -0.519** |
|  | NO_2_ | 0.826** | 0.718** | 0.886** | 0.847** | 1 | -0.601** |
|  | O_3_-8h | -0.454** | -0.244* | -0.636** | -0.519** | -0.601** | 1 |
| Siping |  | PM_2.5_ | PM_10_ | SO_2_ | CO | NO_2_ | O_3_-8h |
|  | PM_2.5_ | 1 | 0.871** | 0.769** | 0.877** | 0.826** | -0.531** |
|  | PM_10_ | 0.871** | 1 | 0.645** | 0.757** | 0.712** | -0.337** |
|  | SO_2_ | 0.769** | 0.645** | 1 | 0.841** | 0.775** | -0.575** |
|  | CO | 0.877** | 0.757** | 0.841** | 1 | 0.831** | -0.539** |
|  | NO_2_ | 0.826** | 0.712** | 0.775** | 0.831** | 1 | -0.574** |
|  | O_3_-8h | -0.531** | -0.337** | -0.575** | -0.539** | -0.574** | 1 |
| Songyuan |  | PM_2.5_ | PM_10_ | SO_2_ | CO | NO_2_ | O_3_-8h |
|  | PM_2.5_ | 1 | 0.830** | 0.808** | 0.612** | 0.806** | -0.452** |
|  | PM_10_ | 0.830** | 1 | 0.686** | 0.518** | 0.662** | -0.294* |
|  | SO_2_ | 0.808** | 0.686** | 1 | 0.723** | 0.802** | -0.474** |
|  | CO | 0.612** | 0.518** | 0.723** | 1 | 0.690** | -0.278* |
|  | NO_2_ | 0.806** | 0.662** | 0.802** | 0.690** | 1 | -0.454** |
|  | O_3_-8h | -0.452** | -0.294* | -0.474** | -0.278* | -0.454** | 1 |
| Suihua |  | PM_2.5_ | PM_10_ | SO_2_ | CO | NO_2_ | O_3_-8h |
|  | PM_2.5_ | 1 | 0.948** | 0.397** | 0.653** | 0.577** | -0.291* |
|  | PM_10_ | 0.948** | 1 | 0.459** | 0.667** | 0.631** | -0.264* |
|  | SO_2_ | 0.397** | 0.459** | 1 | 0.634** | 0.820** | -0.187 |
|  | CO | 0.653** | 0.667** | 0.634** | 1 | 0.692** | -0.398** |
|  | NO_2_ | 0.577** | 0.631** | 0.820** | 0.692** | 1 | -0.343** |
|  | O_3_-8h | -0.291* | -0.264* | -0.187 | -0.398** | -0.343** | 1 |
| Yanbian |  | PM_2.5_ | PM_10_ | SO_2_ | CO | NO_2_ | O_3_-8h |
|  | PM_2.5_ | 1 | 0.945** | 0.804** | 0.660** | 0.851** | -0.250* |
|  | PM_10_ | 0.945** | 1 | 0.708** | 0.605** | 0.786** | -0.127 |
|  | SO_2_ | 0.804** | 0.708** | 1 | 0.570** | 0.680** | -0.372** |
|  | CO | 0.660** | 0.605** | 0.570** | 1 | 0.738** | -0.212 |
|  | NO_2_ | 0.851** | 0.786** | 0.680** | 0.738** | 1 | -0.409** |
|  | O_3_-8h | -0.250* | -0.127 | -0.372** | -0.212 | -0.409** | 1 |
| Changchun |  | PM_2.5_ | PM_10_ | SO_2_ | CO | NO_2_ | O_3_-8h |
|  | PM_2.5_ | 1 | 0.901** | 0.702** | 0.723** | 0.807** | -0.383** |
|  | PM_10_ | 0.901** | 1 | 0.635** | 0.605** | 0.795** | -0.166 |
|  | SO_2_ | 0.702** | 0.635** | 1 | 0.709** | 0.665** | -0.523** |
|  | CO | 0.723** | 0.605** | 0.709** | 1 | 0.742** | -0.431** |
|  | NO_2_ | 0.807** | 0.795** | 0.665** | 0.742** | 1 | -0.285** |
|  | O_3_-8h | -0.383** | -0.166 | -0.523** | -0.431** | -0.285** | 1 |
| ****. Correlation is significant at the 0.01 level.**  ***. Correlation is significant at the 0.05 level.** | | | | | | | |

**Table S3.** The proportions of the six cases.

|  | Case 1 | Case 2 | Case 3 | Case 4 | Case 5 | Case 6 |
| --- | --- | --- | --- | --- | --- | --- |
| proportion | 0.36 | 16.91 | 30.55 | 16.91 | 19.27 | 16.00 |
